# Genome-Wide Comparative Analyses Reveal the Dynamic Evolution of Nucleotide-Binding Leucine-Rich Repeat Gene Family among Solanaceae Plants

**DOI:** 10.3389/fpls.2016.01205

**Published:** 2016-08-10

**Authors:** Eunyoung Seo, Seungill Kim, Seon-In Yeom, Doil Choi

**Affiliations:** ^1^Department of Plant Science, Plant Genomics and Breeding Institute, College of Agriculture and Life Sciences, Seoul National UniversitySeoul, South Korea; ^2^Department of Horticulture, Institute of Agriculture and Life Science, Gyeongsang National UniversityJinju, South Korea

**Keywords:** nucleotide-binding leucine-rich repeat, effector-triggered immunity, Solanaceae, plant innate immune system, genome-wide comparative analysis, resistance genes

## Abstract

Plants have evolved an elaborate innate immune system against invading pathogens. Within this system, intracellular nucleotide-binding leucine-rich repeat (NLR) immune receptors are known play critical roles in effector-triggered immunity (ETI) plant defense. We performed genome-wide identification and classification of NLR-coding sequences from the genomes of pepper, tomato, and potato using fixed criteria. We then compared genomic duplication and evolution features. We identified intact 267, 443, and 755 NLR-encoding genes in tomato, potato, and pepper genomes, respectively. Phylogenetic analysis and classification of Solanaceae NLRs revealed that the majority of NLR super family members fell into 14 subgroups, including a TIR-NLR (TNL) subgroup and 13 non-TNL subgroups. Specific subgroups have expanded in each genome, with the expansion in pepper showing subgroup-specific physical clusters. Comparative analysis of duplications showed distinct duplication patterns within pepper and among Solanaceae plants suggesting subgroup- or species-specific gene duplication events after speciation, resulting in divergent evolution. Taken together, genome-wide analysis of NLR family members provide insights into their evolutionary history in Solanaceae. These findings also provide important foundational knowledge for understanding NLR evolution and will empower broader characterization of disease resistance genes to be used for crop breeding.

## Introduction

Plants and animals have immune systems that protect against invading pathogens, with members of the signal-transduction ATPases with numerous domains (STAND) superfamily of proteins playing important roles in these systems (Maekawa et al., 2011; Duxbury et al., 2016). In plants, the immune system can be divided into two defensive layers (Chisholm et al., 2006; Dangl et al., 2013). The first barrier is composed of cell-surface pattern recognition receptors that recognize conserved pathogen-associated molecular patterns (PAMPs), such as flagellin and chitin. This recognition system provides broad-spectrum, PAMP-triggered immunity (PTI; Zipfel, 2014). To suppress PTI, some pathogens have evolved effector virulence proteins that act in host cells. In turn, plants have a second defensive layer composed of intracellular immune receptors that induce effector-triggered immunity (ETI; Cui et al., 2015). Plants and their pathogens are hypothesized to have evolved attenuating each other (Jones and Dangl, 2006; Fei et al., 2016). Plant genomes contain numerous genes encoding intracellular immune receptors, which either directly or indirectly recognize effectors. Such recognition mediates various downstream defense mechanisms including localized programmed cell death, also known as hypersensitive response (Cui et al., 2015).

Most intracellular immune receptors in plants belong to the nucleotide-binding site and leucine-rich repeat (NLR, also known as NB-LRR) superfamily (Eitas and Dangl, 2010; Lee and Yeom, 2015). They are composed of NB-ARC domains (nucleotide-binding adaptor shared by APAF-1, Resistance genes, and CED-4) and leucine-rich repeat (LRR) domains in central and C-terminal regions, respectively. NLR family proteins are divided into two types based on the presence of a toll and interleukin-1 receptor (TIR) domain in the N-terminus, TIR-NLR (TNL), or absence of this domain, non-TIR-NLR (non-TNL). Some non-TNL proteins have a coiled-coil motif consisting of CC-NLR (CNL). Each domain has consensus motifs with the NB domain that are conserved (Lukasik and Takken, 2009; Yue et al., 2012). In plants, NB-domain proteins are active in ATP binding and hydrolysis, while LRR and N-terminal domains such as TIR and CC are implicated in the activation of, and interaction with, corresponding partners, respectively (Lukasik and Takken, 2009). NB-encoding genes without LRRs may also function in plant immunity (Nandety et al., 2013).

Genome-wide NLR analysis have been pursued in many plants, having been empowered by the accumulation of a variety of genome sequences. Plant genomes show remarkable variation in size and organization, and the numbers of NLRs also vary among plants. For example, 54 NLRs are observed in the papaya genome, while 992 NLRs are present in the apple genome (Porter et al., 2009; Velasco et al., 2010). Variability is observed within the same genus, including 159 and 185 NLRs that are present in *Arabidopsis thaliana* and *A. lyrata*, respectively, and two rice species genomes that have 464 and 483 NLRs, respectively (Yang et al., 2006; Guo et al., 2011). More broadly, monocotyledonous species are known to have fewer TNLs than CNLs (Monosi et al., 2004). Recent studies also show that a pair of neighboring NLRs within the genome may be essential for pathogen resistance targeting transcription factors (Le Roux et al., 2015; Sarris et al., 2015).

Plant NLRs are hypothesized to have co-evolved with pathogens through the course of their natural history together (Jones and Dangl, 2006). Plant NLR homologs are present in extant representatives of early land plants, such as mosses and spikemosses (Xue et al., 2012; Yue et al., 2012). Genome sequencing information and comparative analysis among related species provide some clues on the evolutionary pattern of NLR genes (Yu et al., 2014). In general, NLRs are thought to have undergone rapid evolution that resulted in sequence diversity (Leister, 2004). To date, evolutionary analysis of NLRs have been performed in some crops with available genome data (Jupe et al., 2012; Wan et al., 2013; Lozano et al., 2015).

Solanaceae plants, such as tomato, potato, pepper, and tobacco comprise a large portion of crops worldwide. They are susceptible to various devastating diseases that can result in enormous yield loss (Fry, 2008; Foster and Hausbeck, 2010). Therefore, understanding the molecular mechanisms of disease resistance and the development of resistance to diseases in Solanaceae crops cultivars is essential for success with these crops in agriculture. However, cloning of functional resistance genes via a traditional genetic approach requires much time and effort. To date, about 30 functional resistance genes have been cloned from Solanaceae plants, with most being identified in tomato and potato plants. Genome-wide identification of NLRs represents a potential approach for leading candidate gene cloning and subsequent use of functional NLR genes. Previous studies reported that potato, tomato and pepper genomes have 438, 294, and 684 NLRs, respectively (Jupe et al., 2012; Andolfo et al., 2013; Kim et al., 2014). In addition, recent experiment for capturing NLRs called resistance gene enrichment sequencing (RenSeq) has been conducted in some Solanaceae plants identifying some putative and/or functional NLRs (Jupe et al., 2013; Andolfo et al., 2014; Witek et al., 2016). However, an in-depth comparative analysis of NLR genes across Solanaceae genomes has not yet been achieved.

We identified potential NLR-coding sequences from Solanaceae crops using an in-house pipeline and performed comparative analysis to explore the evolutionary history of the Solanaceae family. We identified 755, 267, and 443 NB-encoding genes from pepper, tomato, and potato, respectively. We also performed phylogenetic and synteny analysis of NLRs in Solanaceae. We found that certain subgroups of NLRs in each species have been expanded and show subgroup-specific duplication patterns after speciation. Our results provide an important blueprint for the identification and characterization of putative R genes, and provide insight into the evolution of NLRs in Solanaceae crops.

## Materials and methods

### Identification and motif analysis of NLR genes in solanaceae genome

NB-encoding genes were identified following previous studies with modification (Meyers et al., 2003; Guo et al., 2011; Xu et al., 2011). Predicted ORFs from reference genomes of tomato (http://solgenomics.net/) and potato (http://solanaceae.plantbiology.msu.edu/) were screened using Hidden Markov model (HMM) search methods (HMMERv.3, http://hmmer.org/) against the pfam NB (NB-ARC) domains (PF00931). NB domains (high quality screened protein set, threshold: 10^−60^) were extracted and used to build a species-specific HMM profile, which was used to identify candidate proteins in each genome by hmmsearch (threshold: 10^−4^; Finn et al., 2011). Then, to identify pepper NLR from pepper genome sequences, all resulting NB domains from tomato and potato were used as queries in tBLASTn searches aimed at finding possible NB-encoding genes in pepper genome (threshold: 10^−4^). All these NB domains were used as queries in BLAST searches for finding possible NB-encoding genes in each genome (threshold: 10^−4^). A total number of 1024 pepper contig sequences contained putative 1312 NLRs. All non-redundant BLAST hits were expanded to 5 kb from both ends of hit, and then the expanded nucleotide sequences were annotated using the gene-prediction pipeline as described in previous study (Kim et al., 2014) to find complete ORFs with removal of ambiguous NLRs containing no start codon, depleted NB domain, and assembly errors. Then, the NB domains were confirmed using pepper specific HMM profile of NB domain with hmmsearch (threshold: 10^−4^) as described above. The annotated NLR genes were validated using BLASTP searches in GenBank to confirm corresponding candidate NLR proteins. These re-annotated pepper NLRs were reflected in PGAv1.55 gene model (http://peppergenome.snu.ac.kr). To further verify TIR, CC, and LRR motifs, candidate NLR proteins were characterized using SMART (http://smart.embl-heidelberg.de/), Pfam database (http://pfam.janelia.org/), and COILS (Lupas et al., 1991) program (threshold = 0.9) following previous work (Kim et al., 2014). Clustering analysis using OrthoMCL was also used for classification of CC motifs (Li et al., 2003).

MEME Suite (Bailey et al., 2009) was used to analyze conserved motifs among Solanaceae NLRs, as previously described (Jupe et al., 2012). A total of 123 NLR sequences were “positive,” including 33 characterized NLRs and 90 predicted randomly selected NLRs. The 90 random NLRs were composed of 15 sequences each from TNL and non-TNL proteins from tomato, potato, and pepper genomes. “Positive” NLRs and “Negative” non-NLRs were used for MEME analysis to identify the 25 most significant motifs (*P* < 10^−4^, having no motif overlap). MAST analysis was then performed to assess the predicted NLRs from each genome, to validate and classify NLRs, and to exclude potential false negatives. The sequences of gene ID identified as NLRs are available at “www.peppergenome.snu.ac.kr” and “www.solgenomics.net.”

### Phylogenetic analyses and classification of solanaceae NLRs

NLR proteins matching the following rule set were selected for phylogenetic analysis as full-type NLRs. Full-type NLRs must have at least: (1) 160 amino acids of NB domain coding sequence, (2) three major motifs (P-loop, kinase, GLPL, or MDHV), and (3) three minor motifs (RNBS-A, RNBS-B, RNBS-C, RNBS-D). Amino acid sequences of NB domains from selected proteins were aligned using ClustalW2 (Larkin et al., 2007), and the alignment was used to construct a phylogenetic tree with the neighbor-joining method using MEGA5 (Tamura et al., 2011). Evolutionary distances were computed using the JTT matrix-based method and 500 sampling repeat-bootstrapping tests were performed. Branches corresponding to partitions that were reproduced in less than half of all bootstrap replicates were collapsed.

To determine subfamilies of Solanaceae NLRs, phylogenetic and clustering analysis were integrated for classification of NLRs in this study (Supplementary Figure 1). Firstly, to define the NLR clusters based on sequence similarities, the OrthoMCL was used. All of identified NLR proteins were obtained with default parameter, and then the pairwise sequence similarities between all NLR protein were estimated by all-by-all BLASTP method (*E*-value cutoff of 1e^−10^ and minimum match length of 50%). A markov clustering algorithm was performed with inflation value of 1.5 (OrthoMCL default parameter). Then, the NLRs in the same cluster were classified as identical subgroups to the phylogenetic subgroups (supported with a bootstrap value higher than 70%). If NLRs in different clusters were the same subgroups from phylogenetic analysis, these were classified as the same subgroup. Finally, NLRs not clustered as singleton (mostly partial and short sequences) were used for BLASTP against classified NLRs and the subgroup was assigned.

### Chromosome location and physical clustering of solanaceae NLRs

NLR-encoding genes (623 of 755, 82.5%) were mapped to their physical position in the genome using pepper pseudomolecules version 1.55 (Kim et al., 2014). For the physical position of NLRs from tomato and potato, general feature format (gff) files of tomato and potato genomes were downloaded from the Sol Genomics Network (https://solgenomics.net/). Visualization of chromosome location was implemented with full-type NLRs using in-house Perl scripts. Physical clustering analysis was performed based on two adjacent NLRs (1) being < 200 kb apart and (2) having fewer than 8 genes between them (Jupe et al., 2012).

### Evolutionary analyses in solanaceae NLRs

Synonymous substitution levels (Ks) between sequences were analyzed. For each set of paralogs the deduced protein sequences were aligned using the Smith–Waterman algorithm (Smith and Waterman, 1981), and the resulting alignment was used as a guide to align respective nucleotide sequences. After removing gaps and N-containing codons Ks were estimated using the maximum likelihood method as implemented in the codeml of the PAML package (Yang, 1997) under the F3 × 4 model (Goldman and Yang, 1994).

A gene family of n members is the result of n-1 gene duplication events. However, the number of possible pairwise comparisons within a gene family [n × (n − 1)/2] can be substantially larger than the number of gene duplications, which can result in multiple estimates for the age of some duplications. To eliminate redundant Ks-values, pairs of duplicated sequences were grouped into gene families using a single-linkage clustering method. A hierarchical clustering method was used to reconstruct the tentative phylogeny of each gene family. Ks-values >2 were discarded from further analysis because they may indicate substitution saturation.

The MCScanX package was implemented for additional duplication and synteny analysis in pepper and tomato genomes (Wang et al., 2012). Protein coding sequences from pepper and tomato genomes were used for all-by-all BLASTP analysis with an *e*-value cutoff of 10^−5^. General feature format (gff) files for physical locations were generated and used for MCScanX.

## Results

### Identification of NLR gene family in solanaceae genome

To compare NLR family genes between Solanaceae plants, we developed an amino acid domain-based pipeline from annotated protein sets and applied it into pepper, tomato, and potato genomic sequences (Supplementary Figure 1). A total of 267, 443, and 755 NB-encoding genes were identified in the genomes of tomato, potato, and pepper, respectively (Table 1). This result was generally consistent with previous studies (Jupe et al., 2012; Andolfo et al., 2013; Kim et al., 2014), but the number of NLRs in the pepper genome increased due to manual curation and improvements of pipeline. The numbers of proteins having both NB and LRR domains were 188, 313, and 422 in the genome of tomato, potato, and pepper, respectively. NB-encoding genes belonging to the CNL group outnumbered those in the TNL group in each of the three Solanaceae genomes analyzed. The pepper genome had many more genes encoding CNL group members compared to tomato and potato, but TNL group numbers were similar across the three genomes. This result was due to the expansion of all types of genes belonging to the CNL group (CC-NLR, NB_cc_-LRR, CC-NB, NB_cc_; Table 1). For example, we found 162 genes having only CNL-type NB domains in pepper, while only 41 and 39 genes were found in tomato and potato, respectively. NB-encoding genes belonging to the TNL group comprised 12, 14.4, and 8.5% of all NB-encoding genes in tomato, potato, and pepper, respectively. These results indicate that Solanaceae NLRs have evolved through a species-specific manner.

**Table 1:** **NLR family proteins in Solanaceae genomes**.

	**Predicted domains^a^**	**Type**	**Pepper**	**Tomato**	**Potato**
NLR type	TIR-NLR	TNL	27	19	37
	CC-NLR	CNL	236	122	177
	NB_tir_-LRR	NL	9	4	9
	NB_cc_-LRR		150	43	90
	Sub-total		422	188	313
NB type	TIR-NB	TN	15	5	9
	CC-NB	CN	143	29	73
	NB_tir_	N	13	4	9
	NB_cc_		162	41	39
	Sub-total		333	79	130
	Total		755	267	443

a *NB, NB-ARC domain; CC, predicted coiled-coils; TIR, Toll interleukin receptor homology; NB_tir_ and NB_cc_, NB-ARC domains derived from those of TNL and CNL, respectively*.

### Phylogenetic analysis and classification of NLRs among solanaceae plants

**Figure 1:**
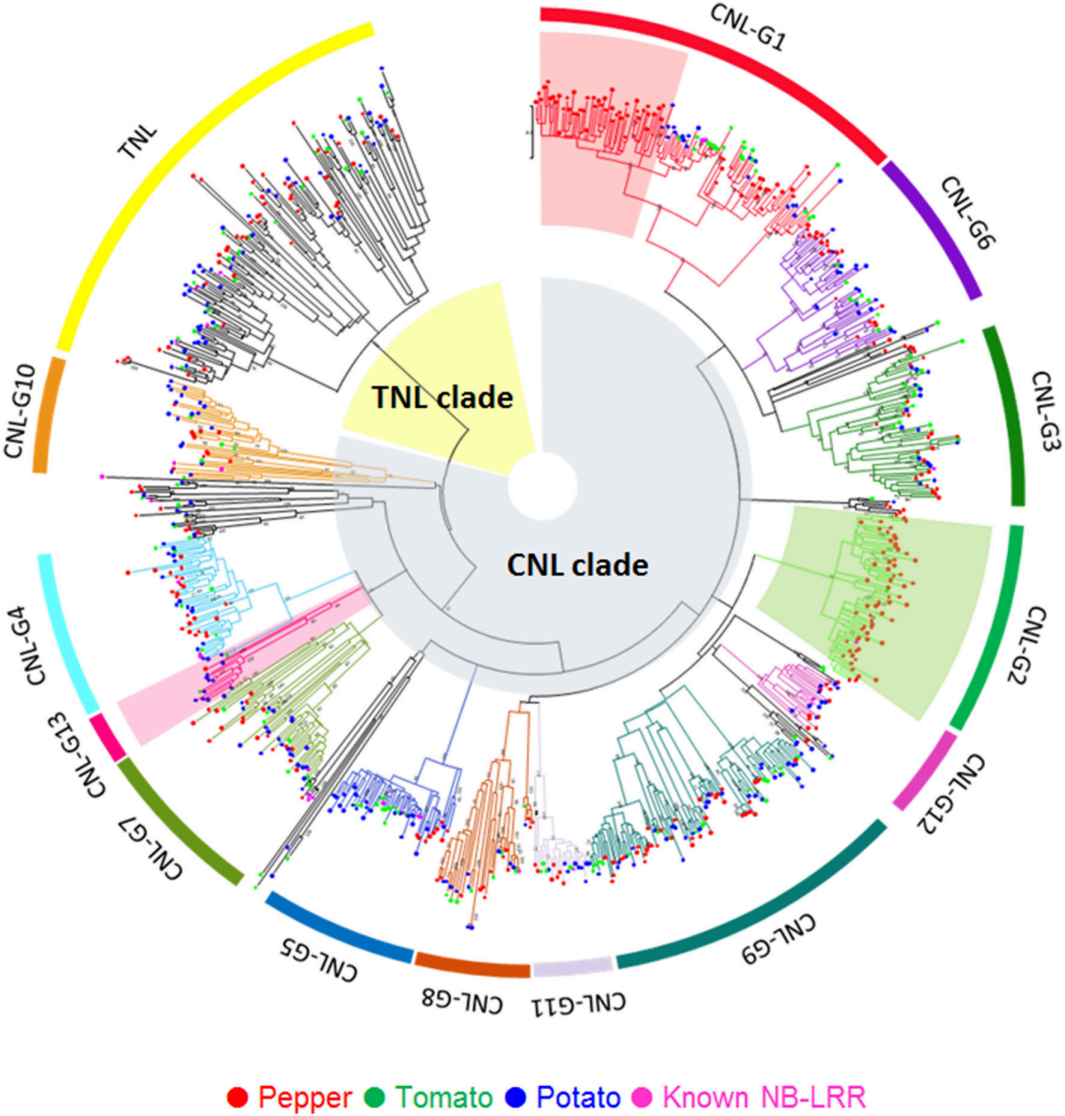
**Phylogenetic relationships of Solanaceae NLRs**. Intact NB domains of tomato (green), potato (blue), and pepper (red), including 31 cloned functional NLR genes (pink) from *Arabidopsis* and Solanaceae species, are used in the construction. The tree is constructed using the neighbor-joining method in MEGA5. Subgroups are classified into 13 CNL types and 1 TNL type. Each color indicates the subgroup and pepper-expanded branches are highlighted with red (CNL-G1) and green (CNL-G2) backgrounds.

For motif analysis, conserved motifs used for MAST searches were built using 123 NLR proteins from Solanaceae, including 33 known R proteins (Jupe et al., 2012). Most were matched to known NLR motifs and some were TNL- or CNL-specific motifs (Supplementary Table 1; Lukasik and Takken, 2009; Yue et al., 2012). To explore the evolutionary relationships among Solanaceae NLRs, intact NB domains were selected based on motif information (see Section Materials and Methods for details). A phylogenetic tree was constructed using the selected NB domain of 791 NLR proteins in Solanaceae, and 31 known R proteins from Arabidopsis and Solanaceae plants (Figure 1). The TNL clade was branched out from the CNL clade, as expected. The CNL clade was divided into 13 additional subgroups. NLR proteins that were removed for tree construction were classified into subgroups based on BLASTP results against assigned NLRs (Table 2 and Supplementary Table 2). All subgroups had at least one NLR protein from tomato, potato and pepper, indicating conservation of NLR classes across Solanaceae genomes, suggesting that all subgroups were present in a common ancestor.

**Table 2:** **Classification of Solanaceae NLRs**.

	**Pepper**		**Tomato**		**Potato**		**Known NLR**	**Potato subgroup^a^**
	**Intact**	**Partial**	**Intact**	**Partial**	**Intact**	**Partial**		
TNL	54	9	26	8	52	12	N, Bs4, Gro1-4, RY-1	TNL
CNL-G1	75	41	25	4	24	12	Rpi-blb2, Hero, Mi-1.2	CNL-1
CNL-G2	91	72	3	1	1	0	Bs2	–
CNL-G3	23	14	14	4	15	2	R1, Prf	–
CNL-G4	32	25	9	8	22	5	L1a, I2, R3a	CNL-8
CNL-G5	10	6	11	13	25	10	R2, Rpi-blb3, Rpi-abpt, R2-like	CNL-5
CNL-G6	26	22	9	5	21	18	Sw-5	–
CNL-G7	18	14	10	5	24	7	Rpi-blb1	CNL-6
CNL-G8	14	5	9	1	13	4	NRC1	–
CNL-G9	48	25	22	11	40	15	–	CNL-3
CNL-G10	34	31	15	15	32	9	RGC2, RPS2, RPS5	CNL-R
CNL-G11	7	3	4	1	10	2	Tm-2a	CNL-4
CNL-G12	9	8	1	1	12	4	Rx, Gpa2	CNL-2
CNL-G13	1	0	1	11	20	5	–	CNL-7
Sub-total	442	275	159	88	311	105		
None-grouping	24	14	19	1	22	5	HRT, RPP8, RCY1, NRG1, RPM1	–
Total	755		267		443			

a *Jupe et al. (2012)*.

However, the numbers of NLRs in certain groups were distinct between species. We found that the numbers of CNL-G1 and CNL-G2 genes were greater than those of other subgroups, with a large portion of NLRs found in pepper (Figure 1). CNL-G1 was composed of 116 genes from pepper, but only 29 and 36 genes from tomato and potato, respectively. Additionally, 163 genes from pepper were classified as CNL-G2, while only 4 genes from tomato and 1 gene from potato were CNL-G2, suggesting that these groups have undergone extensive expansion after the speciation of peppers. Rpi-blb2, Hero, Mi1.2, and CaMi belong to CNL-G1, but Bs2 was the only known R protein in CNL-G2 (Table 2). CNL-G13, previously reported as CNL-7 (Jupe et al., 2012), had expanded in potato based on our findings. These data indicate unequal gene duplication events among subgroups resulting in the observed diversity of the Solanaceae NLR family.

Phylogenetic analysis of Solanaceae NLRs uncovered important information. We found that CaMi, a known pepper R gene, was phylogenetically divergent from other pepper CNL-G1 NLRs and more closely related to those from tomato (Supplementary Figure 2). To determine if CaMi exists in the pepper genome, we mapped the raw sequences from pepper onto a CaMi sequence, but could not find evidence that CaMi was present in pepper genome. However, we found many Mi1.2 homologs in pepper, which could be gene duplication products. Moreover, we built a HMM profile based on the Solanaceae Domain (SD) sequences of six known R proteins (Rpiblb2, Hero, Mi-1.2, R1, Prf, Sw-5) and implemented an hmmsearch in order to find proteins with SDs. SD is a Solanaceae-specific domain located within the extended N-terminus of some Solanaceae CNLs (Mucyn et al., 2006). As a result, we found 94, 35, and 40 NLRs that have a SD in pepper, tomato, and potato, respectively (Supplementary Table 3). Interestingly, all of proteins with SDs belonged to CNL-G1, G3, or G6, and these formed a monophyletic branch even though the phylogenetic tree was constructed using the NB domain (Figure 1). This result indicates that CNL-G1, G3, and G6 could have evolved as Solanaceae-specific clades. In addition, all known R proteins in these subgroups (Rpiblb2, Hero, Mi-1.2, R1, Prf, and Sw-5) have a SD in their N-terminus suggesting that SDs could play important roles in the function of NLRs.

### Genomic localization of NLRs among solanaceae plants

**Figure 2:**
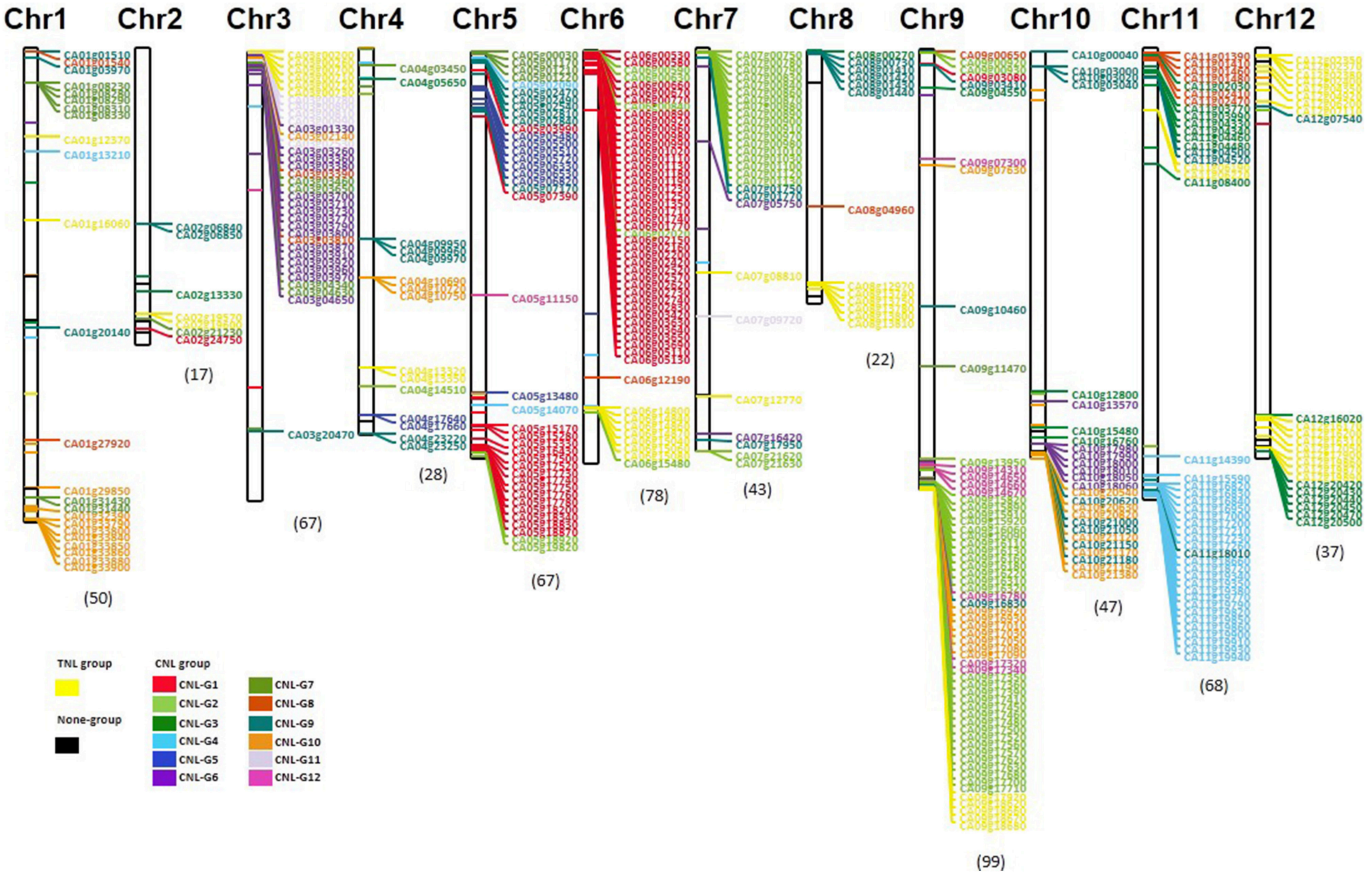
**Physical localization of pepper NLRs**. White rectangular boxes symbolize the 12 pepper chromosomes. Line and letter colors indicate NLR subgroups. For ease of visualization, genes with intact NB domains are represented on the chromosomes. Numbers in parenthesis represent the total numbers of mapped NB-encoding genes on each chromosome.

Genomic location and clustering analysis of NLRs in tomato and potato were performed previously (Jupe et al., 2012; Andolfo et al., 2013). A total of 623 (82.5% of 755) NLR pepper proteins were physically mapped onto all 12 pseudomolecules following the improved annotation of the pepper genome. Among these, NLR gens with an intact NB domain (having at least three major and minor motifs) were represented on chromosomes (Figure 2). Most were positioned at distal chromosome ends, with subgroup members being distributed throughout chromosomes. However, the distribution of NLR genes was not random. CNLs were present on all chromosomes, but TNLs were absent from chromosomes 5 and 10. Chromosome 9 had the highest number of NLR genes (99 genes), with most belonging to CNL-G2. Chromosome 2 had only 17 NLR genes. Most NLRs from the same group were physically clustered by tandem array on specific chromosomes. For example, CNL-G1 NLRs were primarily located on chromosomes 5 and 6, which are closely clustered. These physical clusters might be the result of duplication arising from common ancestors. To investigate localization in-depth, we conducted physical cluster analysis. Based on the physical location of NLRs, we found 111 NLR clusters comprised of 485 genes in the pepper genome by following the same method used for potato NLR cluster analysis (Jupe et al., 2012). These accounted for 77% of all mapped NLRs, with 83 clusters being homogeneously composed of a single subgroup. Most clusters were composed of fewer than 5 genes, but there were 5 clusters composed of more than 10 genes, a result that supported the expansion of NLRs specifically within the pepper genome (Supplementary Figure 3A). The large clusters were located on chromosomes 3, 6, 7, and 9, with the largest cluster on chromosome 9 having 21 CNL-G2 genes and 2 CNL-G12 genes. Of all anchored NLRs in CNL-G2, 94% were clustered (Supplementary Figure 3B).

We compared the distribution of intact NB-encoding genes among pepper, tomato, and potato. As reported, tomato and potato genomes have more conserved synteny of NLRs than did pepper and tomato, or pepper and potato. For example, the proportion of each subgroup on chromosome 4 was similar between tomato and potato genomes (Supplementary Figures 4, 5). However, the organization of NLRs on the upper arm of pepper chromosome 4 was unique. Moreover, potato and pepper showed duplicated NLRs within clusters even though the expanded subgroups were not conserved. On the lower arm of chromosome 9, the pepper CNL-G2 NLRs were clustered together while in potato the CNL-G6 NLRs were clustered together. These results highlight the possibility of species-specific tandem or proximal duplication of NLRs.

### Duplication history among solanaceae NLRs

**Figure 3:**
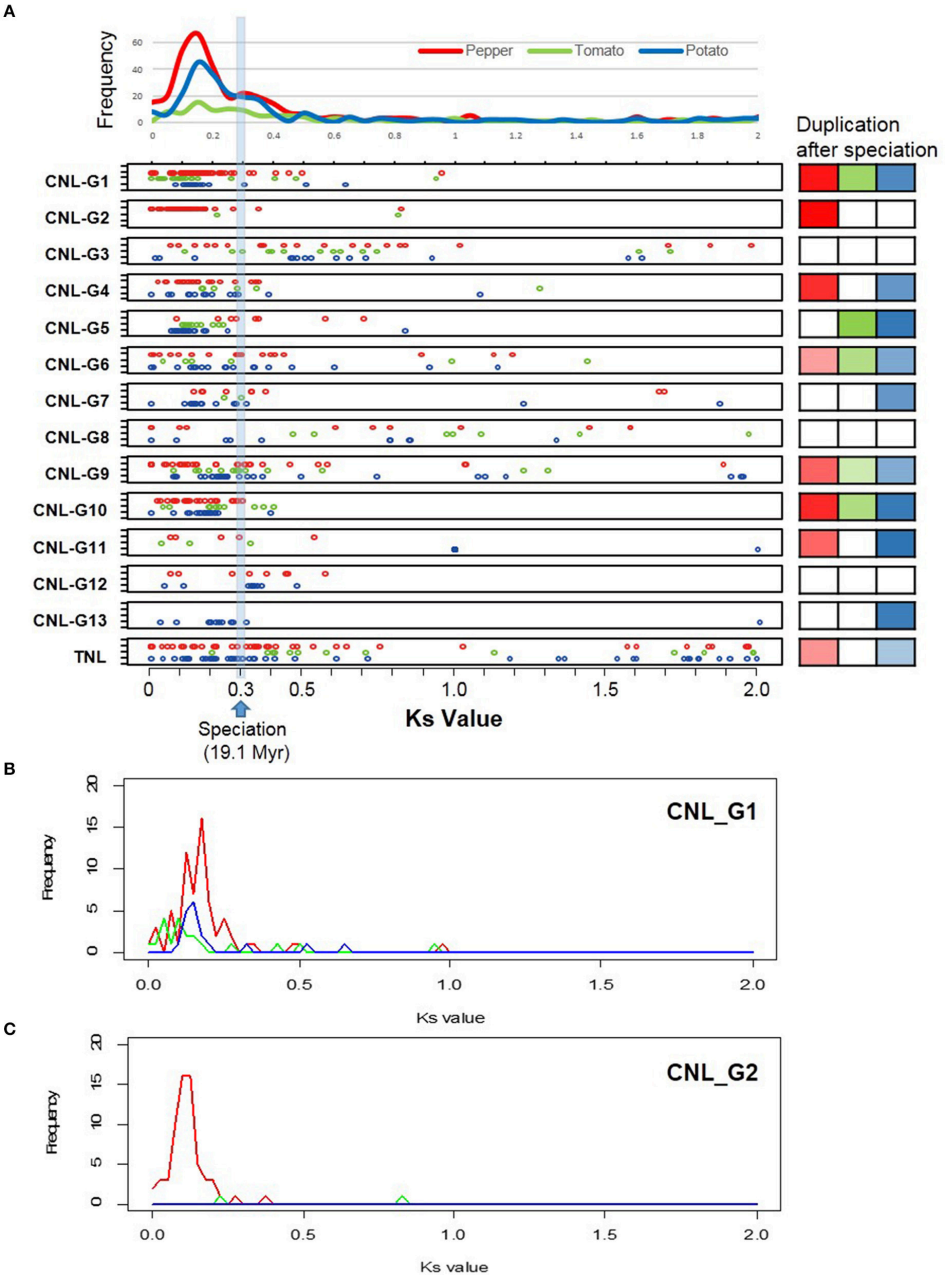
**Duplication history of NLR genes in Solanaceae crop. (A)** Ks-values between paralogs of each subgroup are shown for pepper (red), tomato (green), and potato (blue). X and Y axes represent Ks-values and frequencies, respectively. Estimated speciation time (Ks value of ~0.3) is marked. Significant duplication after speciation is confirmed by chi-square test and is highlighted using colors (right). Intensified colors indicate a high portion of Ks-value after speciation. The distribution pattern of Ks-values for CNL-C1 **(B)** and CNL-C2 **(C)** are shown as examples.

To investigate expansion history of Solanaceae NLRs, gene duplication time was estimated by computing Ks between genes within the same subgroup (Figure 3). The divergence of pepper species from tomato and potato species is estimated to be 19.1 million years ago (Mya), based on a Ks-value of ~0.3 (Kim et al., 2014). As a result, the distribution of Ks-values between NLR paralogs in pepper peaked at ~0.15 indicating that duplication events would have occurred after speciation, around 10 Mya (Figure 3A). In addition, each subgroup showed a specific duplication pattern. We performed a chi-square test and found subgroups that showed a significant duplication increase after speciation. In pepper, CNL-G1, G2, G4, and G10 genes had been amplified dramatically through gene duplication events after speciation. CNL-G1 and G2, pepper-specific expanded groups, showed distinct peaks that indicated expansion by duplication. The peak CNL-G2 Ks-value was ~0.12, while two peaks were observed at ~0.12 and ~0.17 in CNL-G1 (Figures 3B,C).

NLR Ks-values in pepper, tomato, and potato were compared. Peaks from all Ks-values exhibited a similar pattern indicating that duplication of NLRs within each of the three Solanaceae plant types occurred after speciation even though the frequency of duplication differed. However, certain subgroup duplication patterns were unique to specific Solanaceae plants (Figure 3A). For example, distribution patterns of pepper CNL-G1 and G5 were unique when compared to those in tomato and potato (Figure 3B and Supplementary Figure 6). In addition, duplication events in potato CNL-G5 (including R2, Rpi-blb3, and Rpi-abpt) appear to have occurred after the divergence of potato and tomato. Taken together, while the divergence of NLRs from an ancestor before speciation is apparent, unequal gene duplication events after speciation in pepper, or other Solanaceae plants, may lead to different gene repertories in related species.

**Figure 4:**
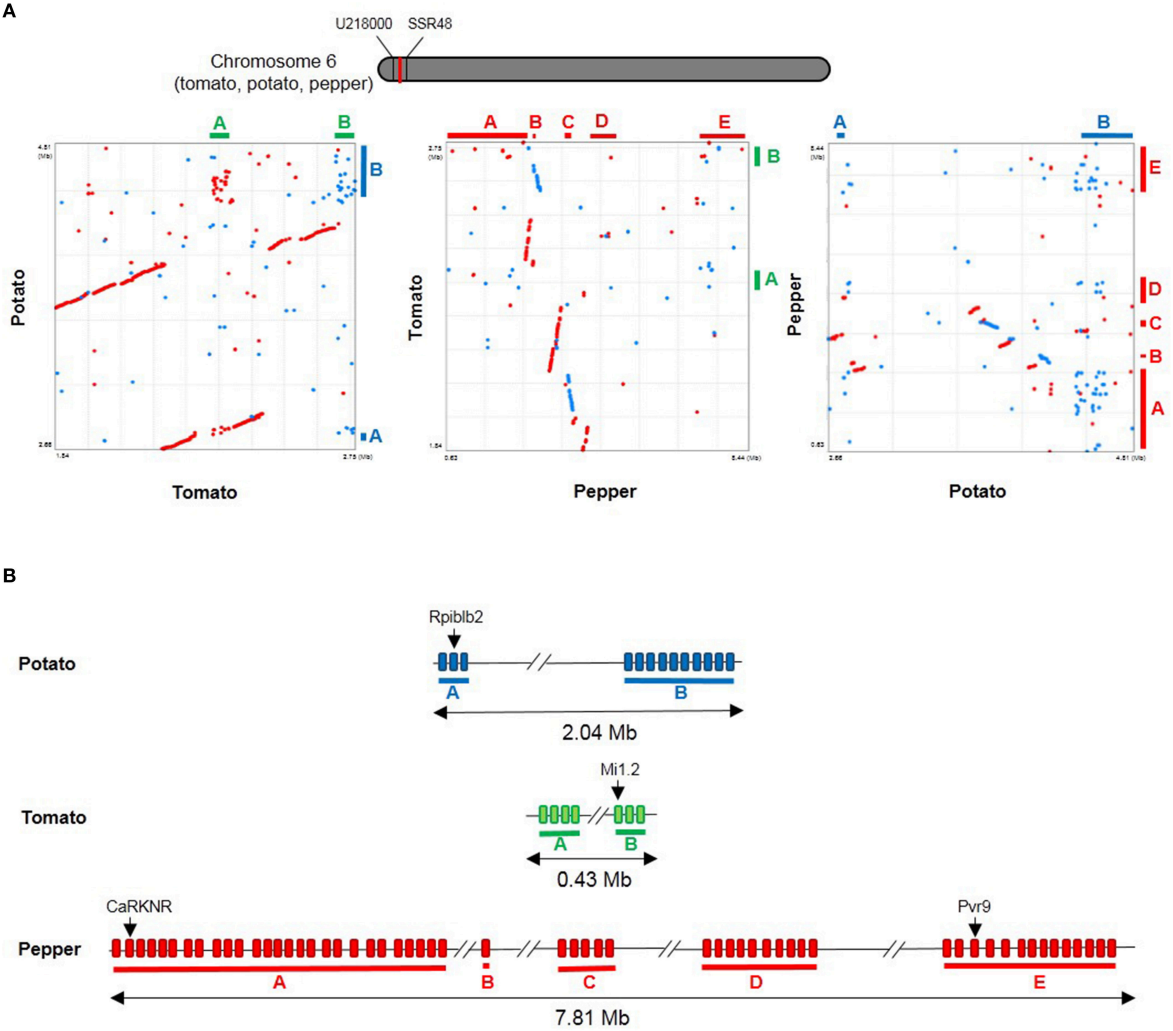
**Comparative analysis of the upper arm of chromosome 6 in Solanaceae. (A)** The region compared in this study is highlighted by a red line on chromosome 6 (top). Flaking markers U218000 and SSR48 from tomato are represented. Dot plots between tomato and potato (left), pepper and tomato (middle), and potato and pepper (right) is presented. Red and blue lines indicate positive and negative alignments, respectively. NLR-rich regions are depicted by lines out of the dot plots. **(B)** The distribution of NLR genes on the upper arm of chromosome 6 is shown. Non-NLR genes were omitted.

The upper arm of chromosome 6 showed many NLRs in pepper and most of them belong to CNL-G1. Pepper chromosome 6 is hypothesized to include many genes related to plant defense response. Pvr9 confers hypersensitive response to *Pepper mottle virus* and is located on chromosome 6 (Tran et al., 2015). CaRKNR is involved in root-knot nematode resistance, and is located on *Capsicum annuum* HDA149 chromosome 6 (Mao et al., 2015). Moreover, a molecular marker study indicates that the dominant source of *Chilli veinal mottle virus* resistance is located on the same chromosome (Lee et al., 2013). This region is known as a hot spot for NLRs in tomato and potato. On the upper arm of tomato chromosome 6, Mi genes are duplicated and clustered (Mi1.1–Mi1.7; Andolfo et al., 2013). Among them, Mi1.2 is known to confer resistance to nematodes, aphids and white flies (Milligan et al., 1998; Rossi et al., 1998; Nombela et al., 2003). In the potato genome, this region includes Rpiblb2, a resistance gene against *Phytophthora infestans* (van der Vossen et al., 2005). We compared this region in pepper, tomato, and potato genomes and observed synteny across species even though there were some rearrangements. However, synteny was not conserved in NLR-rich regions (Figure 4A). We observed expansion in regions with many NLRs, resulting in genome expansion in pepper (Figure 4B). Based on our criteria, 7, 13, and 57 NB-encoding genes were identified in tomato, potato and pepper genomes, respectively, within these regions. Part of the phylogenetic tree in Figure 1 emphasizes the species-specific duplication of pepper NLRs (Supplementary Figure 2). In contrast to the region of tomato showing tandem and proximal duplication of NLRs, most phylogenetically related genes were not in close proximity within the pepper gnome indicating that tandem duplication events alone would not support such an expansion (Supplementary Figure 2). These data suggest that pepper, tomato, and potato have undergone NLR evolution through independent mechanisms and this process resulted in orthologs that target different pathogens.

## Discussion

### Diversification of NLRs among solanaceae plants

Solanaceae is one of the largest families of important crop plants, including tomatoes, potatoes, and peppers. There are a number of pathogens that threaten crop yield, but only a few functional R genes have been cloned. In addition, most cloned R genes are from tomato and potato. The pepper *C. annuum* “Criollo de Morelos-334” (CM334) genome was sequenced recently, and this variety is known to possess various resistance mechanisms against a number of pathogens including *Phytophthora capsici* and *Pepper mottle virus* (Caranta et al., 1999; Truong et al., 2012). We identified and compared NLRs from tomato, potato, and pepper. We used clustering analysis of CC domains to find more CNL-type NLRs. With an improved genome annotation set (version 1.55) and manual curation, we identified 755 genes encoding NB domain in pepper. Among them, 442 genes had both NB and LRR domains, which could represent a pool of candidate resistance genes. Comparisons of the numbers of NLRs in Solanaceae genomes by applying the same approach revealed that pepper genome has more NLRs than tomato and potato (Table 1). However, these numbers are less than that from recently reported study (Wei et al., 2016). The difference of the numbers might be attributed to the standard for identification. We setup the pipeline to remove partial genes and find genes having intact NB domain, which should be used for functional characterization. On the other hand, incomplete sequences matched with only part of NLR genes were counted in previous study (Wei et al., 2016). As results, half of the putative NLR genes from *C. annuum* Zunla were less than 1 kb even though some of them might have introns, which might be pseudogene or trace of NLR duplication.

The NLR gene family is known to be one of the most expanded and variable gene families in plant kingdom. In general, it seems that there is no correlation between the number of NLRs and Solanaceae phylogeny or genome size (Jacob et al., 2013). For example, rice has 458 NLRs while maize has 95 even though they are both monocot plants (Li et al., 2010). In Brassicaceae, the numbers of NLR-encoding genes are similar across species even though these species show variation in genome size and WGD/WGT events (Yu et al., 2014). We found that NLR-encoding genes in pepper were expanded compared to those in tomato and potato. This was evident even though pepper does not show species-specific WGD and despite the fact that the predicted number of total proteins is similar across these species. The observed difference was striking, particularly in the number of CNL-type genes. Taken together, NLRs in Solanaceae might be evolved retaining conserved domains while the total number of genes, and overall types of genes, have diverged.

In pepper, more genes lacking an LRR domain were observed compared to tomato and potato (Table 1). The function of genes belong to types TN, CN, or N is unclear even though they exist in various plant species (Yu et al., 2014). Such genes might serve as reservoirs for diversity or serve to guard other NLRs from genetic aberration. Recent reports show that TX or TN might be involved in plant defense responses (Nandety et al., 2013; Kato et al., 2014; Zhao et al., 2015), but the functions of CN- or N-type proteins remains unclear. Elucidation of their function in plant immunity could provide important insight into plant defense mechanisms.

### Expansion of solanaceae NLR subgroups and features

We used phylogenetic analysis, sequence similarity-based clustering and BLAST search to classify all identified NLRs (see Section Materials and Methods for details). Phylogenetic analysis revealed a high level of conservation across all NLR subgroups even though different numbers of genes were observed in each Solanaceae genome. CNL-type genes were divided into 13 subgroups based on phylogenetic and clustering analysis (Figure 1 and Table 2). Classification of NLRs in Solanaceae revealed that at least one gene from pepper, tomato, and potato from each of the 14 subgroups, including the TNL group. These data indicate that all subgroups were present before Solanaceae speciation. In addition, Solanaceae NLR diversity could be the result of species-specific unequal-duplication events. Phylogenetic analysis also revealed that the expansion of CNL-type genes was primarily due to the expansion of certain subgroups. CNL-G1 and G2 subgroups expanded in pepper, but CNL-G13 expanded in potato. Consistently, results indicated that the divergence of NLRs was derived from ancient progenitors through duplication in Solanaceae.

There are few cloned functional resistance genes from pepper, and these include Bs2, CaMi, and L (Tai et al., 1999; Chen et al., 2007; Tomita et al., 2011). We searched the *C. annuum* CM334 genome for the best homologs to known resistance genes. We found genes that showed more than 90% similarity of NB domain of Bs2 and L, but NB domain of CaMi showed only 75% similarity in the annotated proteins from pepper genome. It is reported that CaMi shares 99% identity in amino acid sequence with tomato Mi1.2 (Chen et al., 2007). However, pepper CNL-G1 genes were unique compared to tomato genes from the same subgroup (Supplementary Figure 2). We mapped raw sequencing reads onto CaMi sequences, but not even the NB domain was covered (data not shown). Based on these results CaMi does not appear to be present in the pepper genome.

We also found some NLRs with a SD. The SD is located before coiled-coil sequences in the N-terminus of some Solanaceae R proteins. This was first reported in the context of Prf and Pto (Mucyn et al., 2006). We found many proteins with SDs and the phylogenetic tree revealed that all belonged to CNL-G1, G3, or G6. We also found six known R proteins that were used to build an HMM profile. We searched for other known R proteins, but did not observe other proteins with SDs (data not shown). Therefore, the SD might have existed before the speciation of Solanaceae and was retained in pepper, tomato, and potato NLRs. The SD of tomato Mi1.2 is reported to play both positive and negative roles in Mi1.2-mediated cell death (Lukasik-Shreepaathy et al., 2012). However, additional functions for the SD have yet to be elucidated. We suggest that the SD plays an important role in Solanaceae immunity and that further in-depth studies using other SD proteins should be conducted.

### Solanaceae NLR physical location and clustering comparisons

Clusters of NLRs are hypothesized to be needed for genetic variation and rapid evolution (Hulbert et al., 2001). In general, R gene clusters are reported in many plant species (Friedman and Baker, 2007; Joshi and Nayak, 2013), and two-thirds of *A. thaliana* or *A. lyrata* NLRs are clustered (Guo et al., 2011). More than 50% of NLR genes are clustered in mammalian genomes even though fewer exist in mammals compared to plants (Jacob et al., 2013). In pepper, 77% of NLRs were clustered in the genome indicating recent tandem or proximal duplication events. The pepper genome has more physical clusters of NLRs than either tomato or potato. In particular, a number of NLRs from the same group were physically clustered even though cluster size varied between subgroups. Still, we found NLR clusters in tomato and potato. Previous research showed that all cloned tomato genes related to resistance are found in clusters or gene arrays (Andolfo et al., 2013). Analysis of clustered NLR genes could provide useful information for genome-guided cloning of functional R genes.

### NLR gene duplications and the evolutionary history in solanaceae

It is common knowledge that plants have undergone genome duplication events. Duplication of NLRs may result in recombinant NLR genes that have novel functions and expression patterns that help counter pathogens (Ashfield et al., 2012). Comparisons of NLRs among Solanaceae plants suggested that expansion and diversification of NLRs contributed to lineage-specific, parallel evolution through unequal gene duplication events. This gave rise to different gene repertoires in closely related species. A burst of gene duplications might have occurred at approximately the time of pepper and tomato/potato divergence. Previously, duplication of NLR genes in tomato and potato was reported (Andolfo et al., 2013). Duplication of NLRs in specific subgroups occurred independently after speciation of pepper, tomato, and potato from their last common ancestor. The explosion of CNL-G1 genes on chromosomes 5 and 6 could be attributed to large-scale segmental duplications followed by local rearrangements.

R clusters facilitate the rapid evolution of novel R gene sequences that have altered specificities (Friedman and Baker, 2007). As noted, CNL-G1 and G2 subgroups showed pepper-specific NLR expansion (Figure 1). In-depth analysis revealed some known plant defense genes on the upper arm of chromosome 6 in the expanded CNL-G1 region. However, duplication patterns were different between pepper, tomato, and potato. These data suggest that the expanded CNL-G1 NLRs might benefit the defense system. Previous studies reporting duplicated genes in clusters presented evidence of different specificities for pathogens as a result of this duplication. For example, I2, R3a, and L are located on a syntenic region of chromosome 4 in tomato, potato, and pepper, respectively. However, they target different pathogens and have diversified functions (Simons et al., 1998; Huang et al., 2005; Tomita et al., 2008). These results underscore the importance of duplicated NLRs. Integration of marker analysis and the genome-wide candidate gene approach can support the efficient identification of functional NLR genes.

Breeding of disease resistant plants and use of resistance genes are essential for reducing yield loss from plant diseases. In this study, we identified NLR-encoding genes in pepper, tomato, and potato genomes. Classification and phylogenetic analysis revealed recent duplication of certain CNL subgroups in pepper and potato. Duplication patterns were subgroup-specific and could have occurred after speciation, resulting in divergent evolution. Our findings provide new insights that will help advance the identification and characterization of novel resistance genes in pepper and other Solanaceae plants.

## Author contributions

ES, SY, and DC conceived the study. ES, SK, and SY analyzed the data. ES and SY drafted the manuscript. All authors read and consented to the final version of the manuscript.

### Conflict of interest statement

The authors declare that the research was conducted in the absence of any commercial or financial relationships that could be construed as a potential conflict of interest.
