# Supplementary material for: Genome-Wide Comparative Analyses Reveal the Dynamic Evolution of Nucleotide-Binding Leucine-Rich Repeat Gene Family among Solanaceae Plants
Source: Front Plant Sci. 2016 Aug 10;7:1205. doi: 10.3389/fpls.2016.01205 (PMC4978739; doi:10.3389/fpls.2016.01205)
Supplement: Supplementary file 1 [file Presentation1.PPTX]

## Slide 1
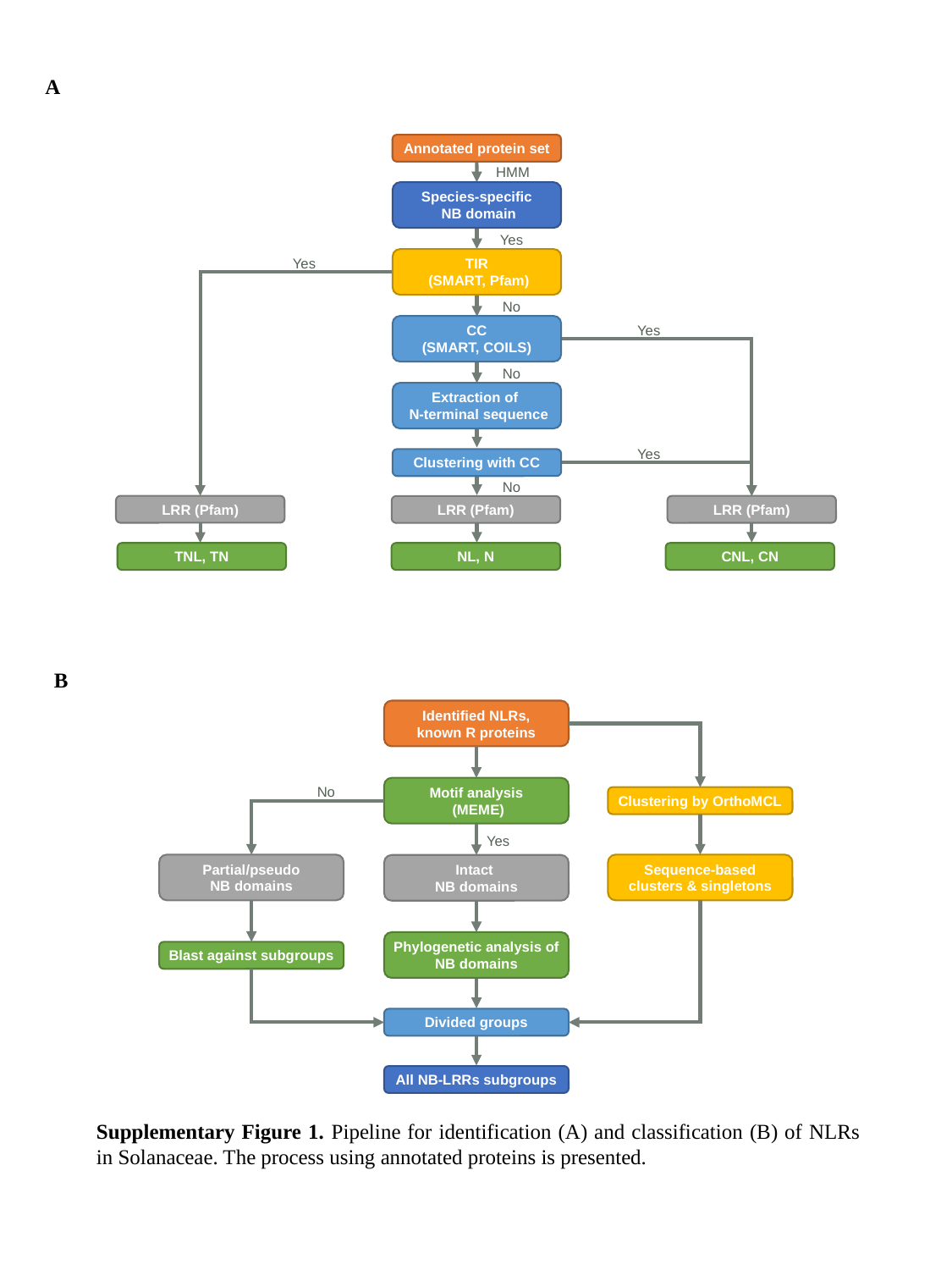

A
Annotated protein set
HMM
Species-specific
 NB domain
Yes
TIR
 (SMART, Pfam)
Yes
No
CC
(SMART, COILS)
Yes
No
Extraction of
 N-terminal sequence
Yes
Clustering with CC
No
LRR (Pfam)
LRR (Pfam)
LRR (Pfam)
TNL, TN
NL, N
CNL, CN
B
Identified NLRs,
known R proteins
Motif analysis
 (MEME)
No
Clustering by OrthoMCL
Yes
Partial/pseudo
NB domains
Sequence-based
clusters & singletons
Intact
NB domains
Phylogenetic analysis of NB domains
Blast against subgroups
Divided groups
All NB-LRRs subgroups
Supplementary Figure 1. Pipeline for identification (A) and classification (B) of NLRs in Solanaceae. The process using annotated proteins is presented.
