# Supplementary material for: Genome-Wide Comparative Analyses Reveal the Dynamic Evolution of Nucleotide-Binding Leucine-Rich Repeat Gene Family among Solanaceae Plants
Source: Front Plant Sci. 2016 Aug 10;7:1205. doi: 10.3389/fpls.2016.01205 (PMC4978739; doi:10.3389/fpls.2016.01205)
Supplement: Supplementary file 2 [file Presentation2.PPTX]

## Slide 1
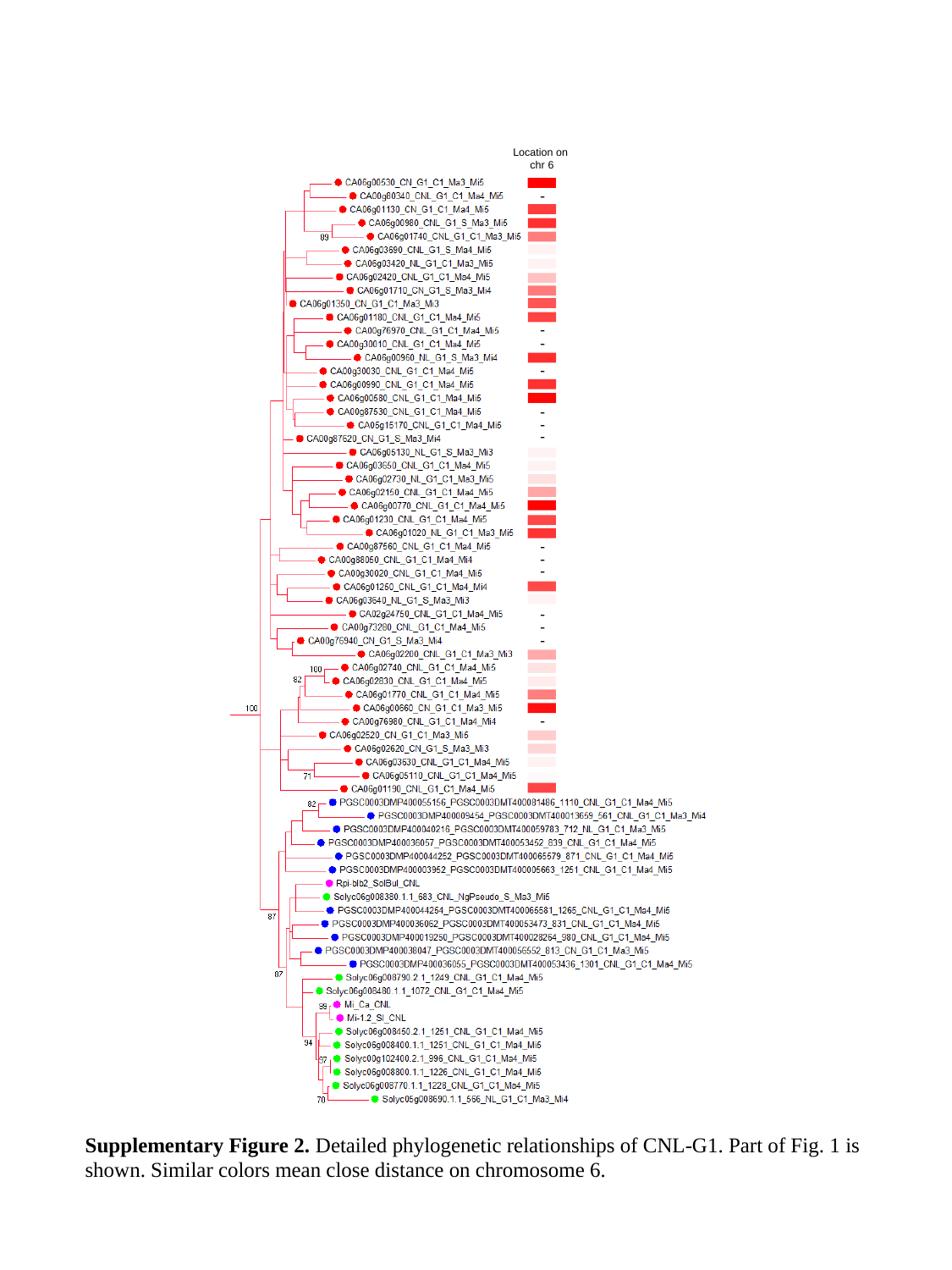

Location on
chr 6
-
-
-
-
-
-
-
-
-
-
-
-
-
-
Supplementary Figure 2. Detailed phylogenetic relationships of CNL-G1. Part of Fig. 1 is shown. Similar colors mean close distance on chromosome 6.
