# Supplementary material for: Genome-Wide Comparative Analyses Reveal the Dynamic Evolution of Nucleotide-Binding Leucine-Rich Repeat Gene Family among Solanaceae Plants
Source: Front Plant Sci. 2016 Aug 10;7:1205. doi: 10.3389/fpls.2016.01205 (PMC4978739; doi:10.3389/fpls.2016.01205)
Supplement: Supplementary file 3 [file Presentation3.PPTX]

## Slide 1
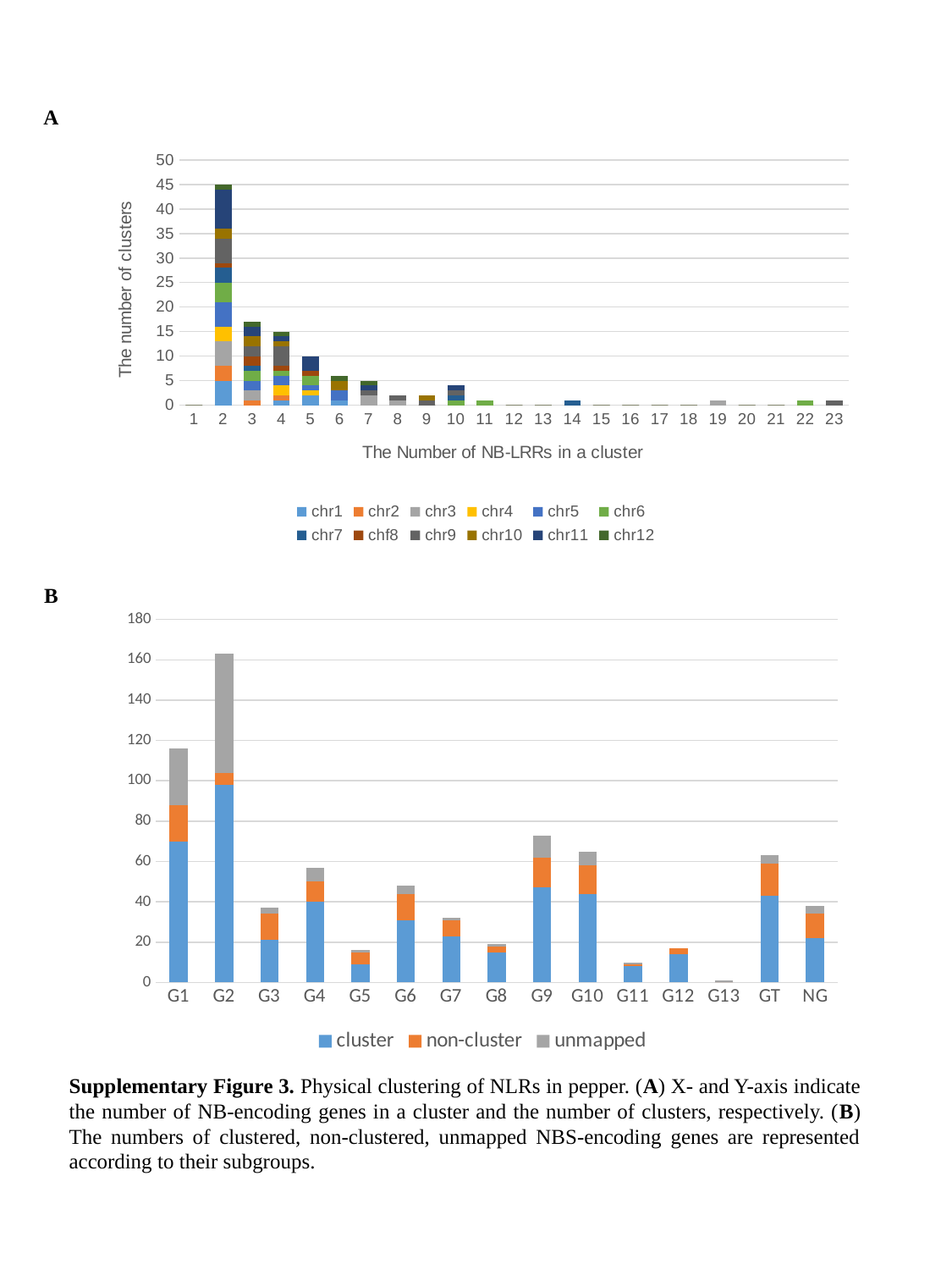

A
### Chart
| Category | chr1 | chr2 | chr3 | chr4 | chr5 | chr6 | chr7 | chf8 | chr9 | chr10 | chr11 | chr12 |
|---|---|---|---|---|---|---|---|---|---|---|---|---|
| 1 | 0.0 | 0.0 | 0.0 | 0.0 | 0.0 | 0.0 | 0.0 | 0.0 | 0.0 | 0.0 | 0.0 | 0.0 |
| 2 | 5.0 | 3.0 | 5.0 | 3.0 | 5.0 | 4.0 | 3.0 | 1.0 | 5.0 | 2.0 | 8.0 | 1.0 |
| 3 | 0.0 | 1.0 | 2.0 | 0.0 | 2.0 | 2.0 | 1.0 | 2.0 | 2.0 | 2.0 | 2.0 | 1.0 |
| 4 | 1.0 | 1.0 | 0.0 | 2.0 | 2.0 | 1.0 | 0.0 | 1.0 | 4.0 | 1.0 | 1.0 | 1.0 |
| 5 | 2.0 | 0.0 | 0.0 | 1.0 | 1.0 | 2.0 | 0.0 | 1.0 | 0.0 | 0.0 | 3.0 | 0.0 |
| 6 | 1.0 | 0.0 | 0.0 | 0.0 | 2.0 | 0.0 | 0.0 | 0.0 | 0.0 | 2.0 | 0.0 | 1.0 |
| 7 | 0.0 | 0.0 | 2.0 | 0.0 | 0.0 | 0.0 | 0.0 | 0.0 | 1.0 | 0.0 | 1.0 | 1.0 |
| 8 | 0.0 | 0.0 | 1.0 | 0.0 | 0.0 | 0.0 | 0.0 | 0.0 | 1.0 | 0.0 | 0.0 | 0.0 |
| 9 | 0.0 | 0.0 | 0.0 | 0.0 | 0.0 | 0.0 | 0.0 | 0.0 | 1.0 | 1.0 | 0.0 | 0.0 |
| 10 | 0.0 | 0.0 | 0.0 | 0.0 | 0.0 | 1.0 | 1.0 | 0.0 | 1.0 | 0.0 | 1.0 | 0.0 |
| 11 | 0.0 | 0.0 | 0.0 | 0.0 | 0.0 | 1.0 | 0.0 | 0.0 | 0.0 | 0.0 | 0.0 | 0.0 |
| 12 | 0.0 | 0.0 | 0.0 | 0.0 | 0.0 | 0.0 | 0.0 | 0.0 | 0.0 | 0.0 | 0.0 | 0.0 |
| 13 | 0.0 | 0.0 | 0.0 | 0.0 | 0.0 | 0.0 | 0.0 | 0.0 | 0.0 | 0.0 | 0.0 | 0.0 |
| 14 | 0.0 | 0.0 | 0.0 | 0.0 | 0.0 | 0.0 | 1.0 | 0.0 | 0.0 | 0.0 | 0.0 | 0.0 |
| 15 | 0.0 | 0.0 | 0.0 | 0.0 | 0.0 | 0.0 | 0.0 | 0.0 | 0.0 | 0.0 | 0.0 | 0.0 |
| 16 | 0.0 | 0.0 | 0.0 | 0.0 | 0.0 | 0.0 | 0.0 | 0.0 | 0.0 | 0.0 | 0.0 | 0.0 |
| 17 | 0.0 | 0.0 | 0.0 | 0.0 | 0.0 | 0.0 | 0.0 | 0.0 | 0.0 | 0.0 | 0.0 | 0.0 |
| 18 | 0.0 | 0.0 | 0.0 | 0.0 | 0.0 | 0.0 | 0.0 | 0.0 | 0.0 | 0.0 | 0.0 | 0.0 |
| 19 | 0.0 | 0.0 | 1.0 | 0.0 | 0.0 | 0.0 | 0.0 | 0.0 | 0.0 | 0.0 | 0.0 | 0.0 |
| 20 | 0.0 | 0.0 | 0.0 | 0.0 | 0.0 | 0.0 | 0.0 | 0.0 | 0.0 | 0.0 | 0.0 | 0.0 |
| 21 | 0.0 | 0.0 | 0.0 | 0.0 | 0.0 | 0.0 | 0.0 | 0.0 | 0.0 | 0.0 | 0.0 | 0.0 |
| 22 | 0.0 | 0.0 | 0.0 | 0.0 | 0.0 | 1.0 | 0.0 | 0.0 | 0.0 | 0.0 | 0.0 | 0.0 |
| 23 | 0.0 | 0.0 | 0.0 | 0.0 | 0.0 | 0.0 | 0.0 | 0.0 | 1.0 | 0.0 | 0.0 | 0.0 |B
### Chart
| Category | cluster | non-cluster | unmapped |
|---|---|---|---|
| G1 | 70.0 | 18.0 | 28.0 |
| G2 | 98.0 | 6.0 | 59.0 |
| G3 | 21.0 | 13.0 | 3.0 |
| G4 | 40.0 | 10.0 | 7.0 |
| G5 | 9.0 | 6.0 | 1.0 |
| G6 | 31.0 | 13.0 | 4.0 |
| G7 | 23.0 | 8.0 | 1.0 |
| G8 | 15.0 | 3.0 | 1.0 |
| G9 | 47.0 | 15.0 | 11.0 |
| G10 | 44.0 | 14.0 | 7.0 |
| G11 | 8.0 | 1.0 | 1.0 |
| G12 | 14.0 | 3.0 | 0.0 |
| G13 | 0.0 | 0.0 | 1.0 |
| GT | 43.0 | 16.0 | 4.0 |
| NG | 22.0 | 12.0 | 4.0 |Supplementary Figure 3. Physical clustering of NLRs in pepper. (A) X- and Y-axis indicate the number of NB-encoding genes in a cluster and the number of clusters, respectively. (B) The numbers of clustered, non-clustered, unmapped NBS-encoding genes are represented according to their subgroups.
