# Supplementary material for: Genome-Wide Comparative Analyses Reveal the Dynamic Evolution of Nucleotide-Binding Leucine-Rich Repeat Gene Family among Solanaceae Plants
Source: Front Plant Sci. 2016 Aug 10;7:1205. doi: 10.3389/fpls.2016.01205 (PMC4978739; doi:10.3389/fpls.2016.01205)
Supplement: Supplementary file 4 [file Presentation4.PPTX]

## Slide 1
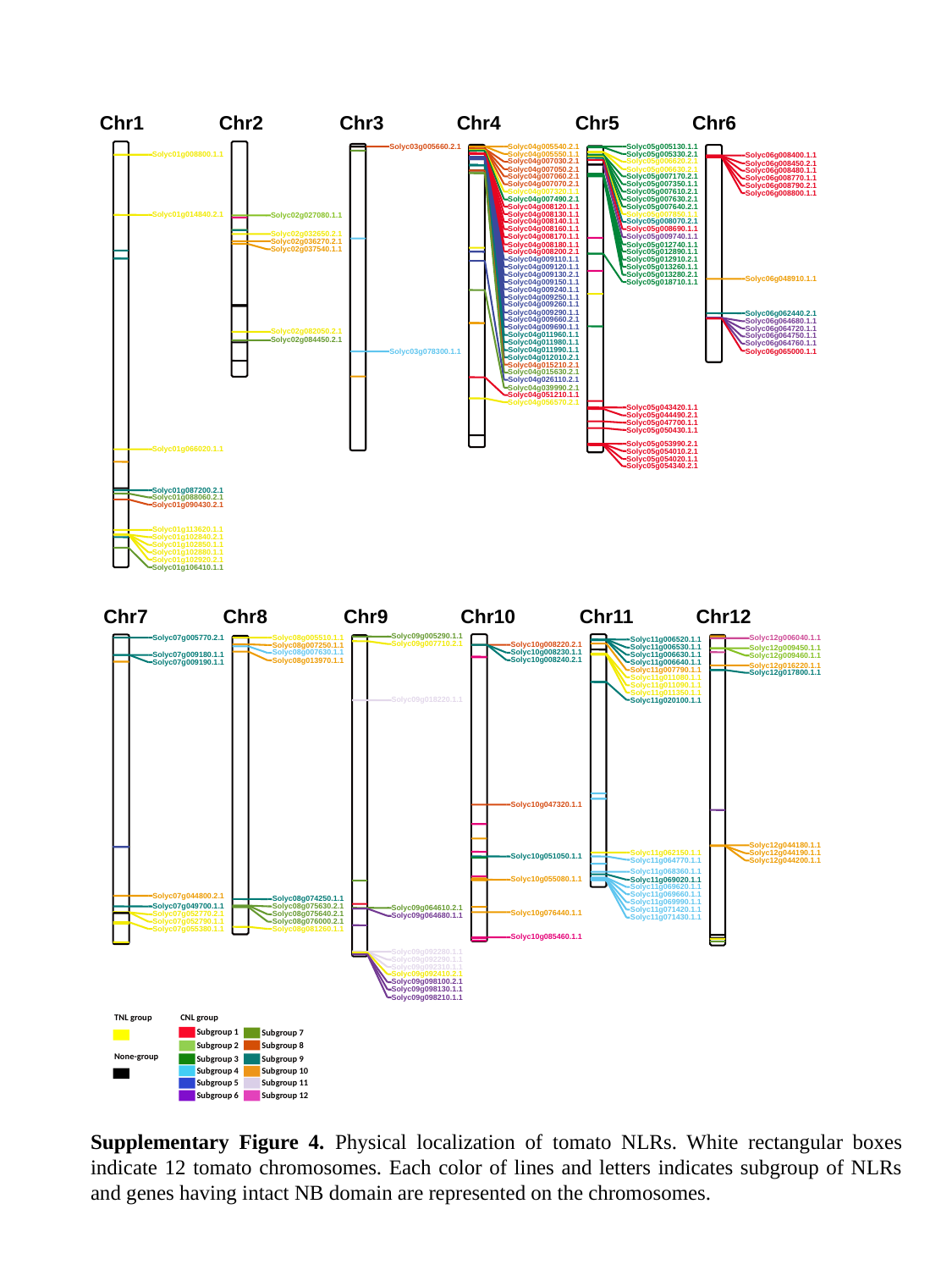

Chr1
Chr2
Chr3
Chr4
Chr5
Chr6
Solyc01g008800.1.1
Solyc01g014840.2.1
Solyc01g066020.1.1
Solyc01g087200.2.1
Solyc01g088060.2.1
Solyc01g090430.2.1
Solyc01g113620.1.1
Solyc01g102840.2.1
Solyc01g102850.1.1
Solyc01g102880.1.1
Solyc01g102920.2.1
Solyc01g106410.1.1
Solyc02g027080.1.1
Solyc02g032650.2.1
Solyc02g036270.2.1
Solyc02g037540.1.1
Solyc02g082050.2.1
Solyc02g084450.2.1
Solyc03g005660.2.1
Solyc03g078300.1.1
Solyc04g005540.2.1
Solyc04g005550.1.1
Solyc04g007030.2.1
Solyc04g007050.2.1
Solyc04g007060.2.1
Solyc04g007070.2.1
Solyc04g007320.1.1
Solyc04g007490.2.1
Solyc04g008120.1.1
Solyc04g008130.1.1
Solyc04g008140.1.1
Solyc04g008160.1.1
Solyc04g008170.1.1
Solyc04g008180.1.1
Solyc04g008200.2.1
Solyc04g009110.1.1
Solyc04g009120.1.1
Solyc04g009130.2.1
Solyc04g009150.1.1
Solyc04g009240.1.1
Solyc04g009250.1.1
Solyc04g009260.1.1
Solyc04g009290.1.1
Solyc04g009660.2.1
Solyc04g009690.1.1
Solyc04g011960.1.1
Solyc04g011980.1.1
Solyc04g011990.1.1
Solyc04g012010.2.1
Solyc04g015210.2.1
Solyc04g015630.2.1
Solyc04g026110.2.1
Solyc04g039990.2.1
Solyc04g051210.1.1
Solyc04g056570.2.1
Solyc05g005130.1.1
Solyc05g005330.2.1
Solyc05g006620.2.1
Solyc05g006630.2.1
Solyc05g007170.2.1
Solyc05g007350.1.1
Solyc05g007610.2.1
Solyc05g007630.2.1
Solyc05g007640.2.1
Solyc05g007850.1.1
Solyc05g008070.2.1
Solyc05g008690.1.1
Solyc05g009740.1.1
Solyc05g012740.1.1
Solyc05g012890.1.1
Solyc05g012910.2.1
Solyc05g013260.1.1
Solyc05g013280.2.1
Solyc05g018710.1.1
Solyc05g043420.1.1
Solyc05g044490.2.1
Solyc05g047700.1.1
Solyc05g050430.1.1
Solyc05g053990.2.1
Solyc05g054010.2.1
Solyc05g054020.1.1
Solyc05g054340.2.1
Solyc06g008400.1.1
Solyc06g008450.2.1
Solyc06g008480.1.1
Solyc06g008770.1.1
Solyc06g008790.2.1
Solyc06g008800.1.1
Solyc06g048910.1.1
Solyc06g062440.2.1
Solyc06g064680.1.1
Solyc06g064720.1.1
Solyc06g064750.1.1
Solyc06g064760.1.1
Solyc06g065000.1.1
Chr7
Chr8
Chr9
Chr10
Chr11
Chr12
Solyc09g005290.1.1
Solyc09g007710.2.1
Solyc09g018220.1.1
Solyc09g064610.2.1
Solyc09g064680.1.1
Solyc09g092280.1.1
Solyc09g092290.1.1
Solyc09g092310.1.1
Solyc09g092410.2.1
Solyc09g098100.2.1
Solyc09g098130.1.1
Solyc09g098210.1.1
Solyc07g005770.2.1
Solyc07g009180.1.1
Solyc07g009190.1.1
Solyc07g044800.2.1
Solyc07g049700.1.1
Solyc07g052770.2.1
Solyc07g052790.1.1
Solyc07g055380.1.1
Solyc08g005510.1.1
Solyc08g007250.1.1
Solyc08g007630.1.1
Solyc08g013970.1.1
Solyc08g074250.1.1
Solyc08g075630.2.1
Solyc08g075640.2.1
Solyc08g076000.2.1
Solyc08g081260.1.1
Solyc12g006040.1.1
Solyc12g009450.1.1
Solyc12g009460.1.1
Solyc12g016220.1.1
Solyc12g017800.1.1
Solyc12g044180.1.1
Solyc12g044190.1.1
Solyc12g044200.1.1
Solyc10g008220.2.1
Solyc10g008230.1.1
Solyc10g008240.2.1
Solyc10g047320.1.1
Solyc10g051050.1.1
Solyc10g055080.1.1
Solyc10g076440.1.1
Solyc10g085460.1.1
Solyc11g006520.1.1
Solyc11g006530.1.1
Solyc11g006630.1.1
Solyc11g006640.1.1
Solyc11g007790.1.1
Solyc11g011080.1.1
Solyc11g011090.1.1
Solyc11g011350.1.1
Solyc11g020100.1.1
Solyc11g062150.1.1
Solyc11g064770.1.1
Solyc11g068360.1.1
Solyc11g069020.1.1
Solyc11g069620.1.1
Solyc11g069660.1.1
Solyc11g069990.1.1
Solyc11g071420.1.1
Solyc11g071430.1.1
TNL group
CNL group
Subgroup 1
Subgroup 7
Subgroup 2
Subgroup 8
Subgroup 3
Subgroup 9
Subgroup 4
Subgroup 10
Subgroup 5
Subgroup 11
Subgroup 6
Subgroup 12
None-group
Supplementary Figure 4. Physical localization of tomato NLRs. White rectangular boxes indicate 12 tomato chromosomes. Each color of lines and letters indicates subgroup of NLRs and genes having intact NB domain are represented on the chromosomes.
