# Supplementary material for: Genome-Wide Comparative Analyses Reveal the Dynamic Evolution of Nucleotide-Binding Leucine-Rich Repeat Gene Family among Solanaceae Plants
Source: Front Plant Sci. 2016 Aug 10;7:1205. doi: 10.3389/fpls.2016.01205 (PMC4978739; doi:10.3389/fpls.2016.01205)
Supplement: Supplementary file 5 [file Presentation5.PPTX]

## Slide 1
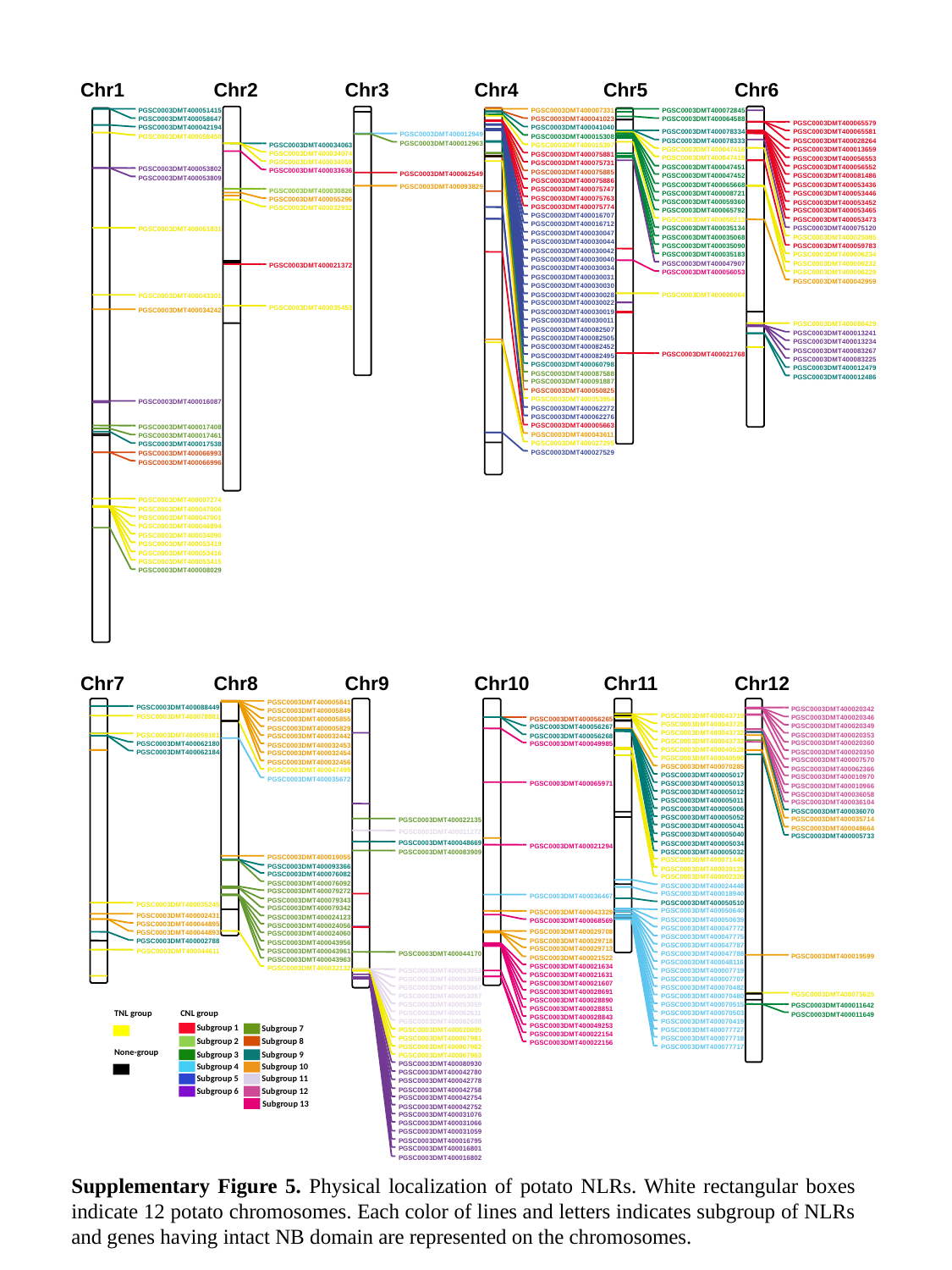

Chr1
Chr2
Chr3
Chr4
Chr5
Chr6
PGSC0003DMT400051415
PGSC0003DMT400058647
PGSC0003DMT400042194
PGSC0003DMT400058450
PGSC0003DMT400053802
PGSC0003DMT400053809
PGSC0003DMT400061831
PGSC0003DMT400043301
PGSC0003DMT400034242
PGSC0003DMT400016087
PGSC0003DMT400017408
PGSC0003DMT400017461
PGSC0003DMT400017538
PGSC0003DMT400066993
PGSC0003DMT400066996
PGSC0003DMT400007274
PGSC0003DMT400047006
PGSC0003DMT400047001
PGSC0003DMT400046894
PGSC0003DMT400034090
PGSC0003DMT400053419
PGSC0003DMT400053416
PGSC0003DMT400053415
PGSC0003DMT400008029
PGSC0003DMT400034063
PGSC0003DMT400034074
PGSC0003DMT400034059
PGSC0003DMT400033636
PGSC0003DMT400030826
PGSC0003DMT400055296
PGSC0003DMT400032932
PGSC0003DMT400021372
PGSC0003DMT400035453
PGSC0003DMT400012949
PGSC0003DMT400012963
PGSC0003DMT400062549
PGSC0003DMT400093829
PGSC0003DMT400007331
PGSC0003DMT400041023
PGSC0003DMT400041040
PGSC0003DMT400015308
PGSC0003DMT400015397
PGSC0003DMT400075881
PGSC0003DMT400075731
PGSC0003DMT400075885
PGSC0003DMT400075886
PGSC0003DMT400075747
PGSC0003DMT400075763
PGSC0003DMT400075774
PGSC0003DMT400016707
PGSC0003DMT400016712
PGSC0003DMT400030047
PGSC0003DMT400030044
PGSC0003DMT400030042
PGSC0003DMT400030040
PGSC0003DMT400030034
PGSC0003DMT400030031
PGSC0003DMT400030030
PGSC0003DMT400030028
PGSC0003DMT400030022
PGSC0003DMT400030019
PGSC0003DMT400030011
PGSC0003DMT400082507
PGSC0003DMT400082505
PGSC0003DMT400082452
PGSC0003DMT400082495
PGSC0003DMT400060798
PGSC0003DMT400087588
PGSC0003DMT400091887
PGSC0003DMT400050825
PGSC0003DMT400053954
PGSC0003DMT400062272
PGSC0003DMT400062276
PGSC0003DMT400005663
PGSC0003DMT400043611
PGSC0003DMT400027295
PGSC0003DMT400027529
PGSC0003DMT400072845
PGSC0003DMT400064588
PGSC0003DMT400078334
PGSC0003DMT400078333
PGSC0003DMT400047416
PGSC0003DMT400047418
PGSC0003DMT400047451
PGSC0003DMT400047452
PGSC0003DMT400065668
PGSC0003DMT400008721
PGSC0003DMT400059360
PGSC0003DMT400065792
PGSC0003DMT400058213
PGSC0003DMT400035134
PGSC0003DMT400035068
PGSC0003DMT400035090
PGSC0003DMT400035183
PGSC0003DMT400047907
PGSC0003DMT400056053
PGSC0003DMT400006064
PGSC0003DMT400021768
PGSC0003DMT400065579
PGSC0003DMT400065581
PGSC0003DMT400028264
PGSC0003DMT400013659
PGSC0003DMT400056553
PGSC0003DMT400056552
PGSC0003DMT400081486
PGSC0003DMT400053436
PGSC0003DMT400053446
PGSC0003DMT400053452
PGSC0003DMT400053465
PGSC0003DMT400053473
PGSC0003DMT400075120
PGSC0003DMT400025085
PGSC0003DMT400059783
PGSC0003DMT400006234
PGSC0003DMT400006232
PGSC0003DMT400006229
PGSC0003DMT400042959
PGSC0003DMT400080429
PGSC0003DMT400013241
PGSC0003DMT400013234
PGSC0003DMT400083267
PGSC0003DMT400083225
PGSC0003DMT400012479
PGSC0003DMT400012486
Chr7
Chr8
Chr9
Chr10
Chr11
Chr12
PGSC0003DMT400088449
PGSC0003DMT400078881
PGSC0003DMT400059181
PGSC0003DMT400062180
PGSC0003DMT400062184
PGSC0003DMT400035245
PGSC0003DMT400002431
PGSC0003DMT400044895
PGSC0003DMT400044893
PGSC0003DMT400002788
PGSC0003DMT400044611
PGSC0003DMT400005841
PGSC0003DMT400005849
PGSC0003DMT400005855
PGSC0003DMT400005829
PGSC0003DMT400032442
PGSC0003DMT400032453
PGSC0003DMT400032454
PGSC0003DMT400032456
PGSC0003DMT400047499
PGSC0003DMT400035672
PGSC0003DMT400019055
PGSC0003DMT400093366
PGSC0003DMT400076082
PGSC0003DMT400076092
PGSC0003DMT400079272
PGSC0003DMT400079343
PGSC0003DMT400079342
PGSC0003DMT400024123
PGSC0003DMT400024056
PGSC0003DMT400024060
PGSC0003DMT400043956
PGSC0003DMT400043961
PGSC0003DMT400043963
PGSC0003DMT400032132
PGSC0003DMT400022135
PGSC0003DMT400011272
PGSC0003DMT400048669
PGSC0003DMT400083909
PGSC0003DMT400044170
PGSC0003DMT400053053
PGSC0003DMT400053056
PGSC0003DMT400053067
PGSC0003DMT400053057
PGSC0003DMT400053059
PGSC0003DMT400062611
PGSC0003DMT400062608
PGSC0003DMT400020005
PGSC0003DMT400067981
PGSC0003DMT400067962
PGSC0003DMT400067963
PGSC0003DMT400080930
PGSC0003DMT400042780
PGSC0003DMT400042778
PGSC0003DMT400042758
PGSC0003DMT400042754
PGSC0003DMT400042752
PGSC0003DMT400031076
PGSC0003DMT400031066
PGSC0003DMT400031059
PGSC0003DMT400016795
PGSC0003DMT400016801
PGSC0003DMT400016802
PGSC0003DMT400056265
PGSC0003DMT400056267
PGSC0003DMT400056268
PGSC0003DMT400049985
PGSC0003DMT400065971
PGSC0003DMT400021294
PGSC0003DMT400036467
PGSC0003DMT400043329
PGSC0003DMT400068569
PGSC0003DMT400029708
PGSC0003DMT400029718
PGSC0003DMT400029713
PGSC0003DMT400021522
PGSC0003DMT400021634
PGSC0003DMT400021631
PGSC0003DMT400021607
PGSC0003DMT400028691
PGSC0003DMT400028890
PGSC0003DMT400028851
PGSC0003DMT400028843
PGSC0003DMT400049253
PGSC0003DMT400022154
PGSC0003DMT400022156
PGSC0003DMT400043719
PGSC0003DMT400043725
PGSC0003DMT400043732
PGSC0003DMT400043733
PGSC0003DMT400040528
PGSC0003DMT400040590
PGSC0003DMT400070285
PGSC0003DMT400005017
PGSC0003DMT400005013
PGSC0003DMT400005012
PGSC0003DMT400005011
PGSC0003DMT400005006
PGSC0003DMT400005052
PGSC0003DMT400005041
PGSC0003DMT400005040
PGSC0003DMT400005034
PGSC0003DMT400005032
PGSC0003DMT400071445
PGSC0003DMT400039125
PGSC0003DMT400002320
PGSC0003DMT400024448
PGSC0003DMT400018940
PGSC0003DMT400050510
PGSC0003DMT400050640
PGSC0003DMT400050639
PGSC0003DMT400047772
PGSC0003DMT400047775
PGSC0003DMT400047787
PGSC0003DMT400047788
PGSC0003DMT400048116
PGSC0003DMT400007719
PGSC0003DMT400007707
PGSC0003DMT400070482
PGSC0003DMT400070480
PGSC0003DMT400070515
PGSC0003DMT400070503
PGSC0003DMT400070419
PGSC0003DMT400077727
PGSC0003DMT400077718
PGSC0003DMT400077717
PGSC0003DMT400020342
PGSC0003DMT400020346
PGSC0003DMT400020349
PGSC0003DMT400020353
PGSC0003DMT400020360
PGSC0003DMT400020350
PGSC0003DMT400007570
PGSC0003DMT400062366
PGSC0003DMT400010970
PGSC0003DMT400010966
PGSC0003DMT400036058
PGSC0003DMT400036104
PGSC0003DMT400036070
PGSC0003DMT400035714
PGSC0003DMT400048664
PGSC0003DMT400005733
PGSC0003DMT400019599
PGSC0003DMT400075625
PGSC0003DMT400011642
PGSC0003DMT400011649
TNL group
CNL group
Subgroup 1
Subgroup 7
Subgroup 2
Subgroup 8
Subgroup 3
Subgroup 9
Subgroup 4
Subgroup 10
Subgroup 5
Subgroup 11
Subgroup 6
Subgroup 12
None-group
Subgroup 13
Supplementary Figure 5. Physical localization of potato NLRs. White rectangular boxes indicate 12 potato chromosomes. Each color of lines and letters indicates subgroup of NLRs and genes having intact NB domain are represented on the chromosomes.
