# Supplementary material for: Genome-Wide Comparative Analyses Reveal the Dynamic Evolution of Nucleotide-Binding Leucine-Rich Repeat Gene Family among Solanaceae Plants
Source: Front Plant Sci. 2016 Aug 10;7:1205. doi: 10.3389/fpls.2016.01205 (PMC4978739; doi:10.3389/fpls.2016.01205)
Supplement: Supplementary file 6 [file Presentation6.PPTX]

## Slide 1
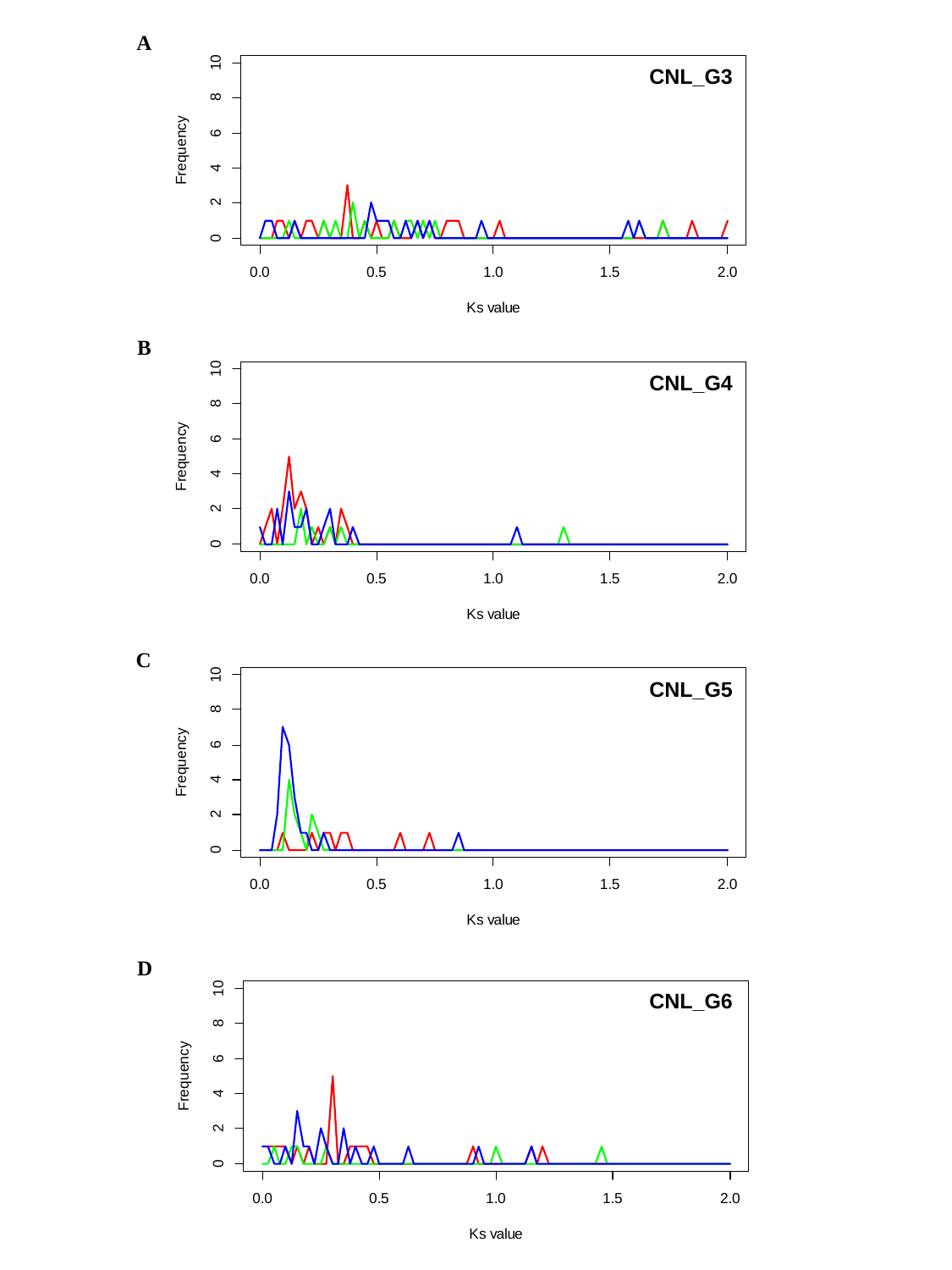

A
CNL_G3
B
CNL_G4
C
CNL_G5
D
CNL_G6

## Slide 2
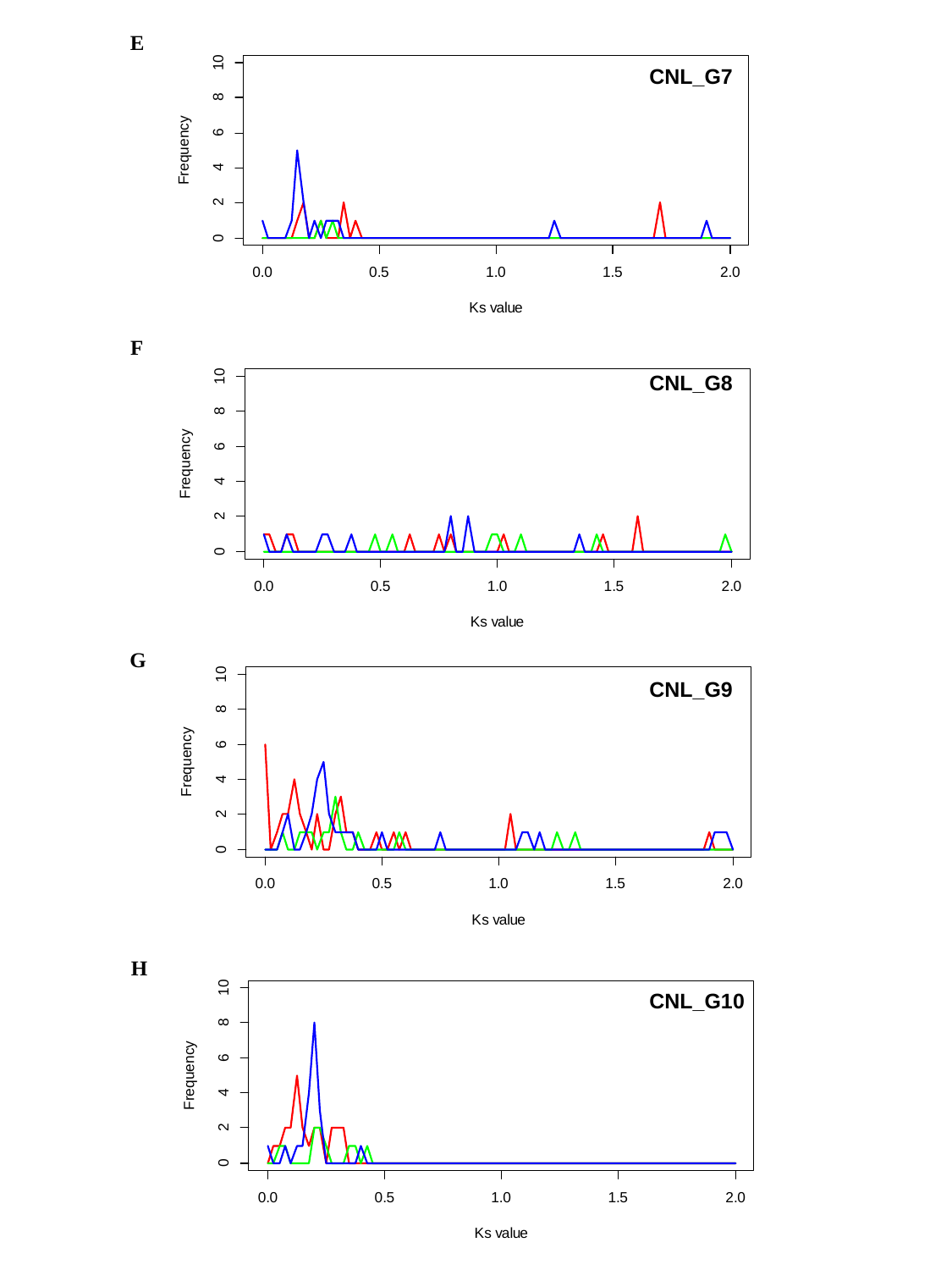

E
CNL_G7
F
CNL_G8
G
CNL_G9
H
CNL_G10

## Slide 3
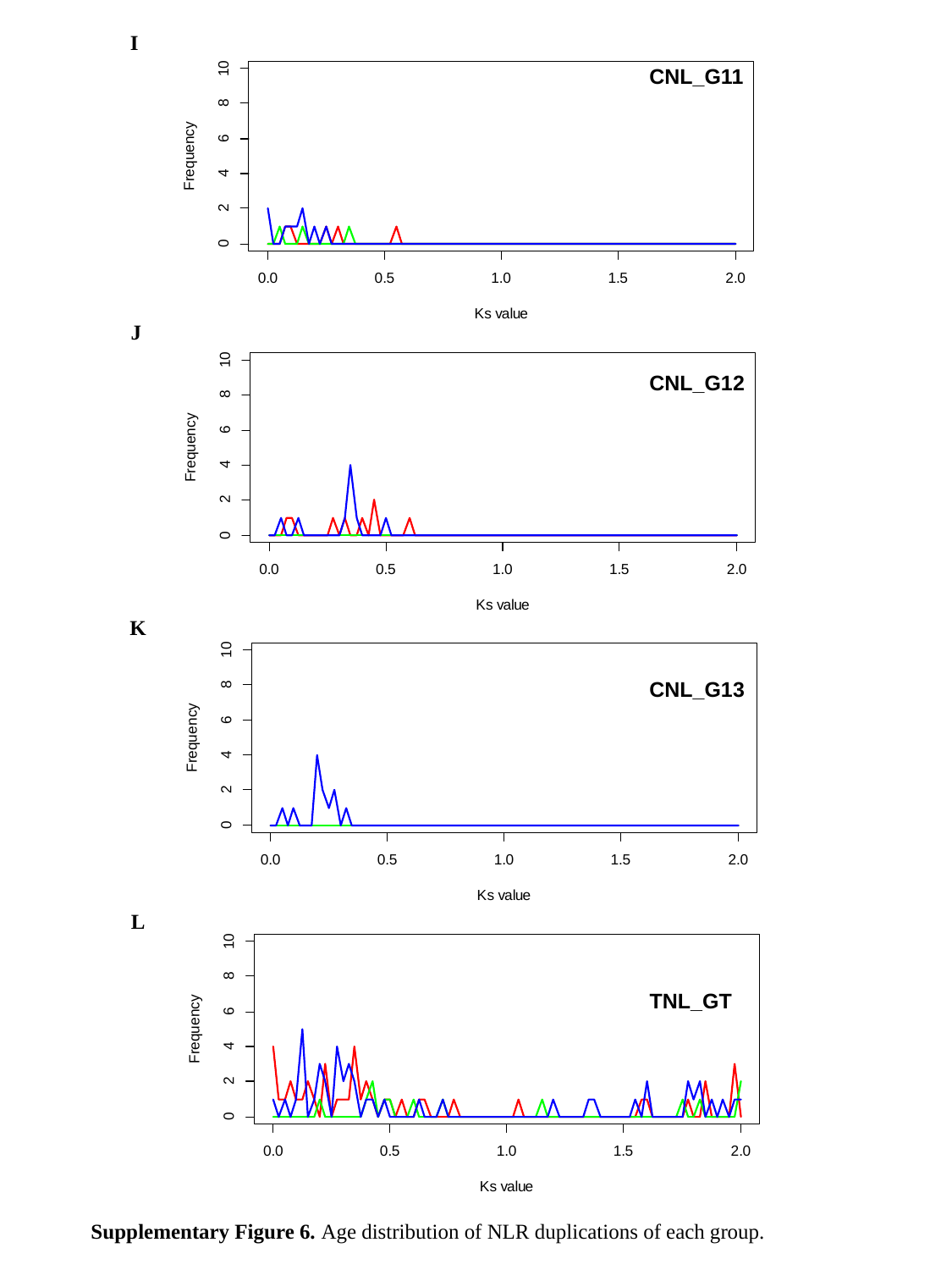

I
CNL_G11
J
CNL_G12
K
CNL_G13
L
TNL_GT
Supplementary Figure 6. Age distribution of NLR duplications of each group.
