# Supplementary material for: Genome-Wide Comparative Analyses Reveal the Dynamic Evolution of Nucleotide-Binding Leucine-Rich Repeat Gene Family among Solanaceae Plants
Source: Front Plant Sci. 2016 Aug 10;7:1205. doi: 10.3389/fpls.2016.01205 (PMC4978739; doi:10.3389/fpls.2016.01205)
Supplement: Supplementary file 7 [file Table1.PDF]

Supplementary Table 1 Major motifs of predicted NB-LRR proteins in Solanaceae species.

Domain	Group	Motif name	Motif ID[a]	Consensus sequence[b]
TIR	TNL	TIR-1	4	WKYDVFLSFRGEDTRKTFTDHLYEALQQKGINTFKDDERLE
		TIR-2	9	LKAIEESRIAIIVFSKNYASSRWCLDELVKIMECK
		TIR-3	8	VLPVFYDVDPSHVRKQKGSFGEAFAKHEER
		TIR-4	15	KVQKWRAALTEAANLSGWDLR
TN linker	TNL		24	DGHESKFIQQIVKDISSKLCR
PreNB	TNL/CNL	preNB	19	VVGIEDExEKIISxLLxGSxD
NB-ARC	TNL/CNL	P-loop	1	VSIVGMGGIGKTTLAKKIYND
	TNL	RNBS-A	25	SQFEGSCFLADVRENSxKxGL
	CNL	RNBS-A	12	HFDVRAWCTVSQEYNERDLLLGILSSISG
	TNL/CNL	Kinase2	3	KGKRYLIVLDDVWDTDQWDDLAGxF
	TNL/CNL	RNBS-B	7	NGSRIILTTRNKEVAEYADxx
	TNL/CNL	RNBS-C	10	YELRLLNDDESWQLFxKKAFG
	TNL/CNL	GLPL	2	LGKEIVKKCKGLPLALKVLGG
	TNL/CNL	-	21	KKTLEEWRSVAExLKSIPxSD
	TNL	RNBS-D	5,18	LKLSYDGLPxHLKPCFLYFACFPEDxxKKDEVTRILESCGFGAEIGIx VLIDKSLI
	CNL	RNBS-D	5,14	LKLSYDGLPxHLKPCFLYFACFPED(x)~9IRLWIAEGFVPxEEEKSLEE VAEEYLEEL
	TNL/CNL	MHD	11	CRMHDLIRDMCRxIARKENFx
NL linker	TNL		16	GKRSRLWDPEDIxEVLxGNTGTEKIEGIS
	TNL/CNL	LRR	23	SAFKKLKNLRVLKLxNxxFxG
	TNL		17	SFEYLPKELRWLSWHGYPLKSLPENFPPE
LRR	TNL/CNL	LRR	6	NLERLVLxxCxNLEEIPxSLGDLxKLxLLNLxNCKKLK
	TNL/CNL	LRR	13	LPSSISKLKNLQTLDVSGCSKLxxLPExL
	TNL/CNL	LRR	20	LPSEIGSLSHLKYLSLS
	TNL/CNL	LRR	22	PSLKYLNLSHSKSLIRTPDFS

[a]Domains and motifs are listed in the order occurred in TNL and CNL proteins.
[b]Consensus amino acid sequence derived from MEME analysis. The red motif names indicate the well-conserved major motifs in NB-ARC domains.
