# Supplementary material for: Genome-Wide Comparative Analyses Reveal the Dynamic Evolution of Nucleotide-Binding Leucine-Rich Repeat Gene Family among Solanaceae Plants
Source: Front Plant Sci. 2016 Aug 10;7:1205. doi: 10.3389/fpls.2016.01205 (PMC4978739; doi:10.3389/fpls.2016.01205)
Supplement: Supplementary file 9 [file Table3.PDF]

Supplementary Table 3. Numbers of NLRs having Solanaceae Domain

	**Pepper**	**Tomato**	**Potato**
CNL-G1	66	24	17
CNL-G3	15	8	10
CNL-G6	13	3	13
Total	94	35	40
